# A PARP1–BRG1–SIRT1 axis promotes HR repair by reducing nucleosome density at DNA damage sites

**DOI:** 10.1093/nar/gkz592

**Published:** 2019-07-10

**Authors:** Yu Chen, Haiping Zhang, Zhu Xu, Huanyin Tang, Anke Geng, Bailian Cai, Tao Su, Jiejun Shi, Cizhong Jiang, Xiao Tian, Andrei Seluanov, Jun Huang, Xiaoping Wan, Ying Jiang, Vera Gorbunova, Zhiyong Mao

**Affiliations:** 1 Clinical and Translational Research Center of Shanghai First Maternity & Infant Hospital, Shanghai Key Laboratory of Signaling and Disease Research, School of Life Sciences and Technology, Tongji University, Shanghai 200092, China; 2 Department of Biology, University of Rochester, Rochester, NY 14627, USA; 3 Life Sciences Institute and Innovation Center for Cell Signaling Network, Zhejiang University, Hangzhou, Zhejiang 310058, China

## Abstract

Creating access to DNA double-strand break (DSB) sites in the chromatin context is an essential step during the repair process, but much remains to be determined about its regulatory mechanisms. Here, using a novel reporter cassette for simultaneous detection of homologous recombination (HR) and nonhomologous end joining (NHEJ) at the same chromosomal site, we report that the efficiency of HR but not NHEJ negatively correlates with nucleosome density. We demonstrate that PARP1 is required for HR by modulating nucleosome density at damage sites. Mechanistic studies indicate that the ATPase domain of BRG1 and the ZnF domain of SIRT1 interact with poly-ADP ribose (PAR) in response to DNA damage, and are responsible for bringing the two factors to broken DNA ends. At DNA damage sites, BRG1 and SIRT1 physically interact, whereupon SIRT1 deacetylates BRG1 at lysine residues 1029 and 1033, stimulating its ATPase activity to remodel chromatin and promote HR.

## INTRODUCTION

Among all types of DNA damage, DNA double strand breaks (DSBs) are the most dangerous. DSBs disrupt the DNA backbone, destabilizing the genome and resulting in deleterious consequences such as tumorigenesis and aging (1–4). Two independent but competing repair pathways, homologous recombination (HR) and nonhomologous end joining (NHEJ), are responsible for repairing DNA DSBs to protect genome integrity (5). In brief, HR is initiated by end resection regulated by the MRN complex and CtIP. The resected single stranded DNA is coated with RPA, followed by the replacement of recombinase RAD51 with the help of several RAD51 paralogs. After copying missing information on sister chromatids, the Holliday junction is resolved by BLM (Sgs1)/TOP3α/RMI1 complex or several other resolvases (6). In contrast, the error-prone NHEJ pathway joins the broken ends with no requirement for homology. Major factors participating in the process include the Ku70/Ku80 heterodimer, DNA-PKcs, Artemis, XRCC4, XLF and DNA Lig 4 (7). The usage rate of the two pathways is determined by many factors such as a cell cycle stage at which cells are damaged (8), the end resection step controlled by the competition between BRCA1/CtIP and 53BP1/Rif1 (9–11).

In mammals, DNA damage and repair occur in the context of chromatin, and chromatin environment surrounding DNA DSBs plays critical roles in DNA damage response and repair (12,13). However, due to the lack of a reporter measuring HR and NHEJ at the same chromosomal site, it has been technically difficult to assess the effect of nucleosome density on the efficiency of HR and NHEJ at the same broken ends.

The ‘access-repair-restore’ model proposes that the chromatin architecture has to be remodeled to allow access to DNA lesions by the DNA repair machinery (14,15). Recent work has indicated that not only in lower eukaryotes such as yeast but also in mammals, multiple chromatin remodeling enzymes are recruited to DNA DSB sites and function at various steps of DNA damage and repair (16–24). By different means, the rapidly recruited CHD4, p400, BRG1 and SNF2H at DNA DSBs facilitate the recruitment of DNA damage signaling proteins such as BRCA1 and 53BP1 (16,18,22,24). Both Ino80 and SCRAP are involved in the step of end resection (17,21). CHD2 stimulates the assembly of NHEJ factors by expanding chromatin and deposing histone variant H3.3 (20). BRG1 interacts with RAD52 to promote the replacement of RPA with RAD51 on single strand DNA to facilitate the process of homology search (19).

PARP1 participates in several types of DNA repair and is an important drug target for cancer therapy (25–27). The recruitment of PARP1 to DNA damage sites is one of the earliest events in the repair process. Previous studies indicated that PARP1 is mainly involved in base excision repair (BER) and single strand break repair (SSB) by recruiting XRCC1, Pol β, Lig 3 and other factors to damaged DNA (28). Recent work has indicated that PARP1 has a similar affinity to additional types of damaged DNA, including blunt DNA ends (29). At DNA DSB sites, PARP1 competes with Ku70 for binding to DNA DSB sites to promote alternative NHEJ (30). In addition, PARP1 is required for the recruitment of CHD2 to DNA DSBs to promote conventional NHEJ (20). Recently, several reports indicate that PARP1 may regulate chromatin remodeling by recruiting the chromatin remodeler ALC1 to DNA lesions to promote nucleotide excision repair (NER) (31–33). However, whether and how PARP1 regulates chromatin density to affect the balance of the two primary DNA DSB repair pathways remains to be further investigated.

Here, we present a new reporter cassette based on the combination of tdTomato and GFP genes with which both HR and NHEJ events can be scored at the same chromosomal locus. Using seven cell lines with single copies of dual reporters integrated at different chromosomal locations, we found that the efficiency of both HR and NHEJ are position dependent, but HR is more sensitive to the nucleosome levels around the DSB site. We demonstrate that PARP1 is required for HR by opening chromatin at DNA damage sites. In response to DNA damage, the rapidly formed PAR recruits BRG1, the ATPase dependent chromatin remodeler, and SIRT1, the deacetylase, to DNA DSB sites, by interacting with ATPase domain of BRG1 and ZnF domain of SIRT1. The recruited BRG1 and SIRT1 interact with one another at DNA damage sites. SIRT1 then deacetylates BRG1 at the lysine residues K1029 and K1033 to activate the BRG1 ATPase activity, therefore stimulating its chromatin remodeling activity to promote chromatin relaxation and HR directed repair.

## MATERIALS AND METHODS

### Cell culture

All fibroblast cell lines were cultured in MEM medium (Hyclone, Cat. #SH30234) supplemented with 10% fetal bovine serum (Life Technologies, Cat. #16000), 1× nonessential amino acid (Hyclone, Cat. #SH3023801) and 1% penicillin/streptomycin (Hyclone, Cat. #SV30010). 293T and Hep3B cells were cultured in DMEM medium (Corning, 10-013-CVR) supplemented with 10% fetal bovine serum (Life Technologies, Cat. #16000), 1 × nonessential amino acid (Hyclone, Cat. #SH3023801) and 1% penicillin/streptomycin (Hyclone, Cat. #SV30010). The cultures were maintained in a 5% CO_2_ and 3% O_2_ humidified incubator (Thermo Fisher Heracell 240i) at 37°C.

### Construction of the reporter cassette

Both parts of the reporter construct were based on the pEGFP-Pem1-Ad2 plasmid (34). On the first part, the ATG-less tdTomato was inserted into the second HindIII recognition site after the first HindIII was removed by Klenow enzyme treatment and self-ligation. On the second part, the first HindIII site was removed and an XhoI site before Ad2 exon was added by site-directed mutagenesis (Strategene, Quikchange Kit, Cat. #200516). Afterwards, the full length of the tdTomato open reading frame was cloned into the XhoI/HindIII sites, replacing the Ad2 exon and second I-SceI recognition site. Then, both the first I-SceI site and XhoI site were mutated. Eventually, the two parts were combined by EcoRI/XhoI digestion and ligation. During the cloning process, all PCRs were performed with the expand long template PCR kit (Roche, Cat. #1681834).

### Generation of HCA2-hTERT and Hep3B reporter cell lines

The reporter cassette linearized at the NheI site was transfected into HCA2-hTERT cells using the Lonza 4D electroporation machine with DT-130 program. At 24-h post transfection, 1 mg/ml G418 was applied to the transfected cells for selection. On days 10–14, colonies were picked and expanded for further analysis. After HCA2-hTERT cell lines containing chromosomally integrated HR-NHEJ reporter cassettes were established, genomic DNA was extracted using the conventional phenol/chloroform method. Real time PCR was performed to determine the copy number using the following primers 5′ CTGACCCTGAAGTTCATCTGCACC 3′, 5′ GAAGAAGTCGTGCTGCTTCATGTG 3′, which amplify part of GFP gene, and 5′ TGGTATGACAACGAATTTGG 3′, 5′ TCTACATGGCAACTGTGAGG 3′, which amplify part of GAPDH gene. Real time PCR reactions were set according to the protocol of FastStart Universal SYBR Green Master Mix (Roche, Cat. No. 04913914001) and run on a Vii7 real-time PCR machine (Life Technologies).

The Hep3B cells were also transfected with the NheI linearized HR-NHEJ reporter construct using Lonza 4D electroporation machine with EH-100 program. At 24-h post transfection, G418 at 1 mg/ml was supplemented to the transfected cells. On day 10 post selection, all Hep3B colonies were pooled together for further analysis of DNA repair efficiency.

### Plasmids and antibodies

The ORF of mTagBFP2 was amplified from pBAD-mTagBFP2 vector purchased from Addgene (34632) and inserted into pEGFP-N1 backbone. Vectors encoding His or GFP tagged full length BRG1 and three separate domains were cloned into pEGFP-N1 backbone after the BRG1 ORF was amplified from the HCA2-hTERT cDNA. Vectors expressing Flag tagged SIRT1 and SIRT1-ΔZnF were created by replacing EGFP with the SIRT1 WT or mutant ORF. All the BRG1 mutants were generated by site-specific mutagenesis method (Transgen, Cat. # FM111-02).

The antibodies used in the study are as follows: HA (Cell signaling, Cat. #2367), PARP1 (Cell signaling, Cat. #46D11), Actin (Santa cruz, Cat. #SC-47778), γH2Ax (Cell signaling, Cat. #9718S), CtIP (Active motif, Cat. #61141), Rad51(Abcam, Cat. #ab179897), His (Abways, Cat. #ab0002), BRG1 (Abcam, Cat. #ab70558), SIRT1 (Millipore, Cat. #07-131), PAR (Trevigen, Cat. #4335-MC-100), Flag (Abclonal, Cat. #AE005), AcK (Abcam, Cat. #ab21623).

### Transfections

All HCA2-hTERT derived cell lines including these harboring reporter cassettes were electroporated with the indicated amount of DNA on a Lonza 4D machine with DT-130 program. For co-IP experiments, an exogenous plasmid encoding tagged BRG1 WT and mutants, SIRT1 and mutants were introduced to 293 cells using P-Pei transfection.

### FACS analysis

On day 3 or day 4 post transfection, cells were harvested and resuspended in 0.3–0.5 ml PBS for FACS analysis on FACS Canto (BD Biosciences) or FACS Verse with a blue 488 laser and violet 405 laser (BD Biosciences). At least 20 000 events were counted. All results were further analyzed using FlowJo software.

### Genome walking

The integration sites were identified as described in the manual of a genome walking kit (Takara, Cat. #6108). According to the supplier's description, three specific primers recognizing known regions of reporter cassette, CCACCTCTGACTTGAGCGTCGATT, ACCGCCATGCATTAGTTATTAATTGA, GGCACACTAGTTGTTTTACCCTAAAG, were designed. Three rounds of PCR were performed. The conditions of each PCR reactions are as follows. First round of PCR: 94°C 1 min; 98°C 1 min; 94°C 30 s, 65°C 1 min, 72°C 3 min (5 cycles); 94°C 30 s; 25°C 3 min; 72°C 3 min; 94°C 30 s, 65°C 1 min, 72°C 3 min, 94°C 30 s, 65°C 1 min, 72°C 3 min, 94°C 30 s, 44°C 1 min, 72°C 3 min (15 cycles); 72°C 10 min. The conditions of the second and third rounds of PCR are the same: 94°C 30 s, 65°C 1 min, 72°C 3 min, 94°C 30 s, 65°C 1 min, 72°C 3 min, 94°C 30 s, 44°C 1 min, 72°C 1 min (15 cycles); 72°C 10 min. After PCR reactions were complete, the amplified specific bands were cloned into a TA vector (Transgen, Cat. # CT301) for further sequencing with M13F and M13R primers.

### Calculation of relative nucleosome density

On day 2 post splitting, at least 1 million cells were harvested and stored at −80°C overnight. On the next day cells were thawed on ice and lysed in 5 ml NP-40 lysis buffer (10 mM Tris–HCl pH 7.4, 10 mM NaCl, 3 mM MgCl_2_, 0.5% NP-40, 0.15 mM spermine, 0.5 mM spermidine) for 5 min at 4°C. The pellet was washed by 2.5 ml ice-cold MNase digestion buffer (10 mM Tris–HCl pH 7.4, 15 mM NaCl, 60 mM KCl, 0.15 mM spermine, 0.5 mM spermidine), and re-suspended in 100 μl MNase digestion buffer containing 1 mM CaCl_2_ before the lysate was incubated with 800 units of MNase (NEB, M0247S) for 5 min at room temperature. The reaction was stopped with 20 μl MNase stop buffer (100 mM EDTA, 10 mM EGTA pH 7.5) and 80 μl MNase digestion buffer. Then the DNA was extracted using conventional phenol/chloroform assay. The relative nucleosome density was then calculated as described (35). In brief, real-time PCR was performed using FastStart DNA Master SYBR Green Mix (Roche) on a ViiA 7 Real-Time PCR system (Applied Biosystems). Data was analyzed using the 2^− ΔΔCt^ method. The PCR primers for quantifying the relative nucleosome density at R1, R2, R3, R4, R5 regions and partial region of GAPDH are as follows:

R1-F, 5′AGCTGTACAAGTAAAGCGGCCGCG3′, R1-R, 5′ATTTGTAACCATTATAAGCTGCAA3′; R2-F, 5′AGGCTATTCGGCTATGACTGGGCA3′, R2-R, 5′CCCGTCGTGGCCAGCCACGATAGC3′; R3-F, 5′AGCAACGCGGCCTTTTTACGGTTC3′, R3-R, 5′TTATAACCCAAATGCTGCCTGTTG3′; R4-F, 5′CTGAGAGCCCTTTTCATCTTTGCT3′, R4-R, 5′GGAAGGGGCAGCAATGAGTTGA3′; R5-F, 5′CCTTTGAATACCTGCCTCTTACTC3′, R5-R, 5′CACCGCAACCAGCCTCAATA3′; GAPDH-F, 5′TGGTATGACAACGAATTTGG3′, GAPDH-R, 5′TCTACATGGCAACTGTGAGG3′.

### Co-immunoprecipitation

All co-IP experiments were performed using 293 cells. At 24 h post splitting or transfection, cells were harvested for lysing with lysis buffer (20mM HEPES pH 8.0, 0.2 mM EDTA, 5% glycerol, 150mM NaCl, 1% NP40). The lysate was incubated on ice for 10 min, followed by sonication on ice at 50% duty for 5 s, and the lysate was then centrifuged at 13 000 rpm for 1 min at 4°C. The supernatant was collected for preclearing with 50% protein A and IgG antibody for 1 hour at 4°C. After centrifuging at 8000 rpm for 1 min at 4°C, the supernatant was collected and added with antibodies at concentrations as the suppliers suggested. After overnight incubation, protein A sepharose was added to the lysate followed by rotating at 4°C for 1 h. After washing 4–5 times with lysis buffer, 2× sample buffer was added and boiled for 10 min. Then the supernatant was collected for further western blot analysis or Mass spec analysis (Hangzhou PTM-biolabs, China).

### Immunofluorescence

The immunostaining experiments were performed as previously reported (36). Cells were cultured on coverslips and fixed with 4% paraformaldehyde for 15 minutes at room temperature. Then the fixed cells were permeabilized with 0.25% Triton-X100 for 10 min. Afterward, the detergent was washed away with PBS for three times with 10 min every time. Cells were then blocked with 1% goated serum for 1 h at room temperature, followed by overnight incubation with primary antibodies. After three time washes with PBS, the secondary antibody was added for 1 h incubation at room temperature. Samples were then washed three times and stained with DAPI for 2 min, followed by another three PBS washes. In the end, the slides were covered with mounting medium (Vector Laboratories, USA) and pictures were taken on a scanning laser microscopy (Leica, USA).

### ChIP assay

The ChIP assay was performed using the NHEJ-I9a cell line as previously reported (36,37). In brief, to quantify the recruitment of SIRT1, BRG1 or their mutants to DSB sites, vectors encoding tagged full length SIRT1 and BRG1 or their mutants were transfected into NHEJ-I9a cells along with I-SceI expression vectors. At 2-h post transfection of vectors encoding Flag-SIRT1 and Flag-SIRT1 mutant, or at 8-h post transfection of vectors encoding GFP-BRG1 and GFP-BRG1 mutants, cells were harvested and chromatin immunoprecipitation was carried out using an antibody against Flag (Sigma-Aldrich, F3165) or GFP (Chromotek, gta-20). DNA from precipitated samples and input was used as the template for real-time PCR with following primers ChIP-F, 5′TGCTTGCCTTGGCTTC AGTG3′ and ChIP-R, 5′CTTGGAAACACCCATGTTGAAATATC3′ on a ViiA 7 Real-Time PCR system (Applied Biosystems).

### Protein purification

The ORF of BRG1 and its derived mutants tagged with His were cloned into the pEGFP-N1 backbone and transfected to exponentially growing 293F cells. Forty-eight hours post transfection, cells were harvested and frozen-thawed three times in liquid nitrogen and 37°C water bath. The His tagged proteins were then purified from the lysate using the Nickel beads (GE, Cat. #17-3712-01) according to the description provided by the manufacturer.

### *In vitro* co-IP assay

Purified His tagged protein from 293F (full-length BRG1, BRG1-N, ATPase, BRG1-C, SIRT1 or SIRT1-ΔZnF) were incubated with biotin-labeled PAR (Trevigen, Cat. # 4336-100-02) for 2 h at 4 °C. Then the streptavidin beads (CST, Cat. #5947S) were added to the mixture for an additional 2 h. The beads were washed with PBST for three times, then were boiled in sample buffer for 10 min. Samples were analyzed by western blot with an antibody against His.

### *In vitro* deacetylation assay

The reactions for analyzing deacetylation were performed as previously reported (38). The reactions contained 10 mM Tris–HCl, 150 mM NaCl, 10% glycerol, 0.8 mM NAD+ and were incubated at 30°C for 2 h before being subjected for western blot analysis.

### *In vitro* ATPase assay

The ATPase activity was measured using ATPase/GTPase activity assay kit (Sigma, Cat. #MAK113). Briefly, the BRG1 WT or mutant was mixed with the assay buffer (40 mM Tris, 80 mM NaCl, 8 mM MgAc_2_, 1 mM EDTA pH 7.5) supplemented with 4mM ATP. The mixture was incubated at 30°C for 2 h and before the reaction was terminated with 200 μl malachite green reagent. The absorbance was read at 620 nm after 20 min incubation at room temperature on the Eon microplate spectrophotometer (Biotek, USA).

### *In vitro* nucleosome sliding assay

The nucleosomes were packaged *in vitro* on a 247-bp rDNA fragment by PCR from mouse genomic DNA according to salt dialysis method. The nucleosome sliding assay was described as previously reported (31,39). The packaged nucleosomes, BRG1 WT or mutant and additional proteins were incubated at 30°C for 2 h in the reaction buffer containing 20 mM HEPES pH 7.9, 50 mM NaCl, 4.5 mM MgCl_2_, 2 mM DTT, 0.5 mM PMSF, 45 μg/ml BSA, 10% glycerol, 0.02% Triton X-100, 0.02% NP-40 and 2 mM ATP. Then the mixture was run on 4.5% native polyacrylamide gel and stained in ethidium bromide for 20 min before the pictures were taken.

### Clonogenic assay

Cells were pretreated with increasing concentrations of PARP1 inhibitors in six-well plates for 24 h. Then cells were supplemented with etoposide at a different concentration ranging from 0 to 0.5 μM. On day 10 post the drug treatment, cells were stained with commassie regent (0.25% Commassie, 50% methanol and 10% acetic acid) and colonies with at least 50 cells were counted.

## RESULTS

### Construction and validation of dual-fluorescent reporter cassette

**Figure 1.**
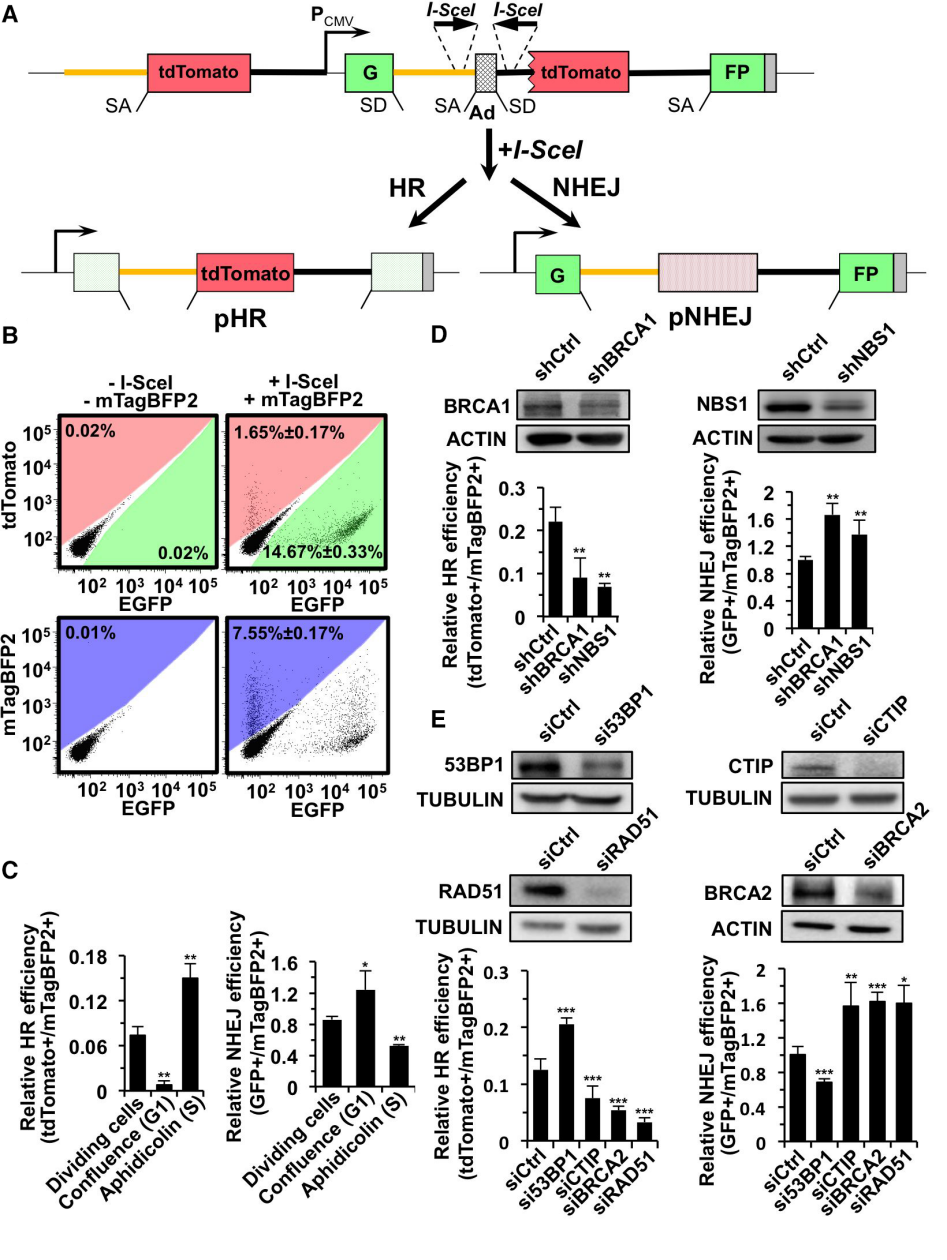
Construction and validation of the novel HR-NHEJ reporter cassette for simultaneous detection of HR and NHEJ at the same genomic locus. (**A**) Diagram of the dual fluorescent reporter substrate (HR-NHEJ reporter). The HR-NHEJ reporter consists of two parts, separated by the CMV promoter. The part downstream of the promoter contains a GFP gene interrupted by an engineered rat Pem1 intron, an adenoviral exon (AD2) flanked by two I-SceI recognition sites in an inverted orientation and an ATG-less tdTomato gene. The second part, upstream of the promoter, contains a Pem1 intron inserted with a full length tdTomato gene. After digestion by the I-SceI restriction enzyme, the Ad2 exon will be removed. HR directed repair leads to pHR, which contains a GFP gene separated by the Pem1-intron and a full-length tdTomato gene, which will result in a functional tdTomato gene fused to the first GFP exon. Successful repair by NHEJ results in pNHEJ, containing a functional GFP gene. Yellow line, the first half of the rat Pem1 intron before the AD2 exon or tdTomato gene; dark line, the second half of the rat Pem1 intro after the Ad2 exon and ATG-less tdTomato or tdTomato gene. The yellow line represents 5′ homologous sequence, and tdTomato ORF is the 3′ homologous sequence. SD, splice donor. SA, splice acceptor. (**B**) Calibration of FACS protocol for simultaneous analysis of HR and NHEJ efficiency using HCA2-D4a cell line harboring a single copy of reporter cassette. No fluorescent signal was observed when a control vector pHPRT-CAG32 was transfected into D4a cells. Co-transfection with vectors encoding I-SceI and pCMV-mTagBFP2 indicated amounts of GFP+, tdTomato+ and mTagBFP2+ cells were detected. (**C**) Validation of the reporter by analyzing the change of HR and NHEJ efficiency in G1 stage and S stage. D4a cells were arrested in G1 or S phase by confluency or aphidicolin treatment as previously reported (8), before transfections and FACS analysis were performed. (D, E) Effect of depleting DNA repair factors on the balance between NHEJ and HR. (**D**) Depleting either BRCA1 or NBS1 impairs HR while promotes NHEJ. (**E**) Knocking down 53BP1 tips cells toward HR from NHEJ, while CtIP, BRCA2 or RAD51 depletion impairs HR and promotes NHEJ. All experiments were repeated at least three times. Error bars indicate s.d. * *P* < 0.05, ** *P* < 0.01, *** *P* < 0.001.

To study the interplay between the two DSB repair pathways and the chromatin context in which repair is taking place, we designed a reporter cassette allowing for detection of both HR and NHEJ-mediated repair events at the same chromosomal sites (Figure 1A). The reporter construct consists of two parts separated by a CMV promoter. The part downstream of the promoter contains a GFP gene interrupted by an engineered rat Pem1 intron, an adenoviral exon (AD2) flanked by two I-SceI recognition sites in an inverted orientation and an ATG-less tdTomato gene. The second part, upstream of the CMV promoter, contains a Pem1 intron inserted with a full length tdTomato gene. Upon the induction of DNA DSBs in response to I-SceI digestion, the Ad2 exon will be removed. HR reconstitutes G-Pem1-full length tdTomato-FP, named product of HR (pHR), while NHEJ restores G-Pem1-ATG-less tdTomato-FP, resulting in NHEJ repair product (pNHEJ) (Figure 1A). At the steps of post-transcriptional modification, due to the existence of a splicing donor (SD) at the junction of G-Pem1 and a splicing acceptor (SA) at the junction of Pem1-tdTomato in pHR, the partial GFP exon is spliced into the full-length tdTomato gene. In contrast, due to the lack of SA before ATG-less tdTomato ORF and the existence of SD at the junction of G-Pem1 and SA at the junction of Pem1-FP in pNHEJ, successful NHEJ restores functional GFP during splicing. To validate the construct design, we first created plasmids encoding pHR and pNHEJ repair products (Figure 1A) and tested if transiently transfected pHR would turn cells red while pNHEJ would turn cells green. As expected, after 5 μg pHR was transfected into the HCA2-hTERT cells, a normal human foreskin fibroblast line immortalized by the ectopic expression of the catalytic subunit of hTERT (40), ∼40% of the cells became red fluorescent. Similarly, when 5 μg of pNHEJ was transfected, ∼50% cells became green fluorescent (Supplementary Figure S1A). The proportion of aberrant splicing events resulting in green fluorescence for HR and red for NHEJ was very small: 0.13% and 0.01% respectively. Thus, over 99.5% of either HR or NHEJ products were correctly represented by red fluorescence or green fluorescence.

To further validate that the HR-NHEJ reporter cassette simultaneously measures HR and NHEJ, we integrated the cassette into HCA2-hTERT cells. After selection with G418 at 1 mg/ml for 10 days, all colonies were pooled together and cells were infected with adenovirus bearing I-SceI gene. We successfully observed both tdTomato+ and GFP+ cells on day 5 post virus infection (Supplementary Figure S1B). Using the same approach, we integrated the dual reporter cassette into the genome of the HCA2-hTERT cells and isolated individual colonies. To obtain cell lines harboring a single copy of the reporter cassette in the genome, we performed real-time PCR using NHEJ-I9a, which is a clone of the HCA2-hTERT cell bearing a single copy of NHEJ reporter cassette, confirmed by Southern blot (41), as a control. We then created 17 individual colonies with single copy of the HR-NHEJ reporter cassette at different chromosomal loci (Supplementary Figure S1C), and we mapped the integration sites for 12 of the cell lines using genome walking method, and confirmed that the integration occurred at different genomic loci (Supplementary Table S1).

We calibrated the FACS analysis by transfecting the D4a cells, a clone of the HCA2-hTERT cells integrated with the dual-fluorescent reporter, with I-SceI vector, together with a blue fluorescent vector encoding mTagBFP2 in order to normalize transfection efficiency (Figure 1B). We also ruled out the possibility that mTagBFP2 expression may interfere with the detection of tdTomato or GFP (Supplementary Figure S1D-E). We therefore used the ratio of tdTomato+/mTagBFP2+ as a measure of HR efficiency, and GFP+/mTagBFP2+ cells as a measure of NHEJ efficiency.

Since the choice of the two pathways is dependent on cell cycle stages (8), we therefore validated the D4a cells by comparing the efficiency of the two pathways in cells arrested in G1 or S stage (Supplementary Figure S2A). We found that HR was nearly absent in G1 cells but stimulated by ∼2-fold in S cells in comparison to that in control cells, while NHEJ was dominant in G1 stage (Figure 1C). These data were consistent with our previous report using HCA2-hTERT cells harboring only HR or NHEJ reporter cassette (8). However, even in S stage, most of the cells seemed to choose NHEJ rather than HR to repair DNA DSBs. We hypothesized that the terminally differentiated fibroblasts probably prefer NHEJ over HR, and the method of electroporation to deliver the exogenous I-SceI vector might cause stresses which affect the pathway choice.

We further validated the D4a cell line by analyzing the change of HR and NHEJ efficiency in response to BRCA1, NBS1, CTIP, BRCA2, RAD51 or 53BP1 depletion by shRNA or siRNA (Figure 1D-E). We found that in agreement with previous studies knocking down either BRCA1, NBS1, CTIP, BRCA2 or RAD51 significantly inhibited HR but promoted NHEJ while depleting 53BP1 led to a significant reduction of NHEJ, and an increase of HR (Figure 1D-E) (5). Moreover, inhibiting MRE11 activity with Mirin led to an over 90% reduction in HR efficiency (Supplementary Figure S2B).

In theory, the HR-NHEJ reporter cassette measures the efficiency of both canonical NHEJ (c-NHEJ) and alternative NHEJ (alt-NHEJ). However, the loss of one sub-pathway for joining the broken ends may be compensated by the other sub-pathway (30,42,43). Therefore, to further validate that the HR-NHEJ reporter measures the efficiency of both c-NHEJ and alt-NHEJ, we knocked down the core c-NHEJ factor DNA-PKcs or/and the key alt-NHEJ component PARP1 in cells harboring the HR-NHEJ reporter (Supplementary Figure S2C), and examined the NHEJ efficiency. We found that knocking down DNA-PKcs or PARP1 alone led to a mild reduction in NHEJ efficiency by 21.6% or 20.7% while knocking down both DNA-PKcs and PARP1 suppressed NHEJ efficiency by ∼ 51% (Supplementary Figure S2C). In addition, we also knocked down DNA-PKcs or/and POLθ, another essential alt-NHEJ factor (44) (Supplementary Figure S2D). In consistence with the PARP1 depletion experiments, we observed that depleting DNA-PKcs or POLθ mildly suppressed NHEJ efficiency by 19.8% or 17.3% while knocking down both DNA-PKcs and POLθ caused a dramatic reduction in NHEJ efficiency by 52.6% (Supplementary Figure S2D). Moreover, in Hep3B cells containing chromosomally integrated HR-NHEJ reporter cassette, inhibiting DNA-PKcs with Nu7026 or PARP1 with olaparib alone mildly suppressed the NHEJ efficiency by 18.6% or 20.3% (Supplementary Figure S2E). In contrast, combining Nu7026 and olaparib caused a significant reduction in NHEJ efficiency by 62.9% (Supplementary Figure S2E). Collectively, these data indicated that the HR-NHEJ reporter measures the efficiency of both c-NHEJ and alt-NHEJ.

Taken together, our results demonstrate that we successfully created the dual reporter cassette for analyzing the efficiency of both HR and NHEJ at the same chromosomal site.

### HR but not NHEJ is sensitive to nucleosome density at DNA DSB sites

**Figure 2.**
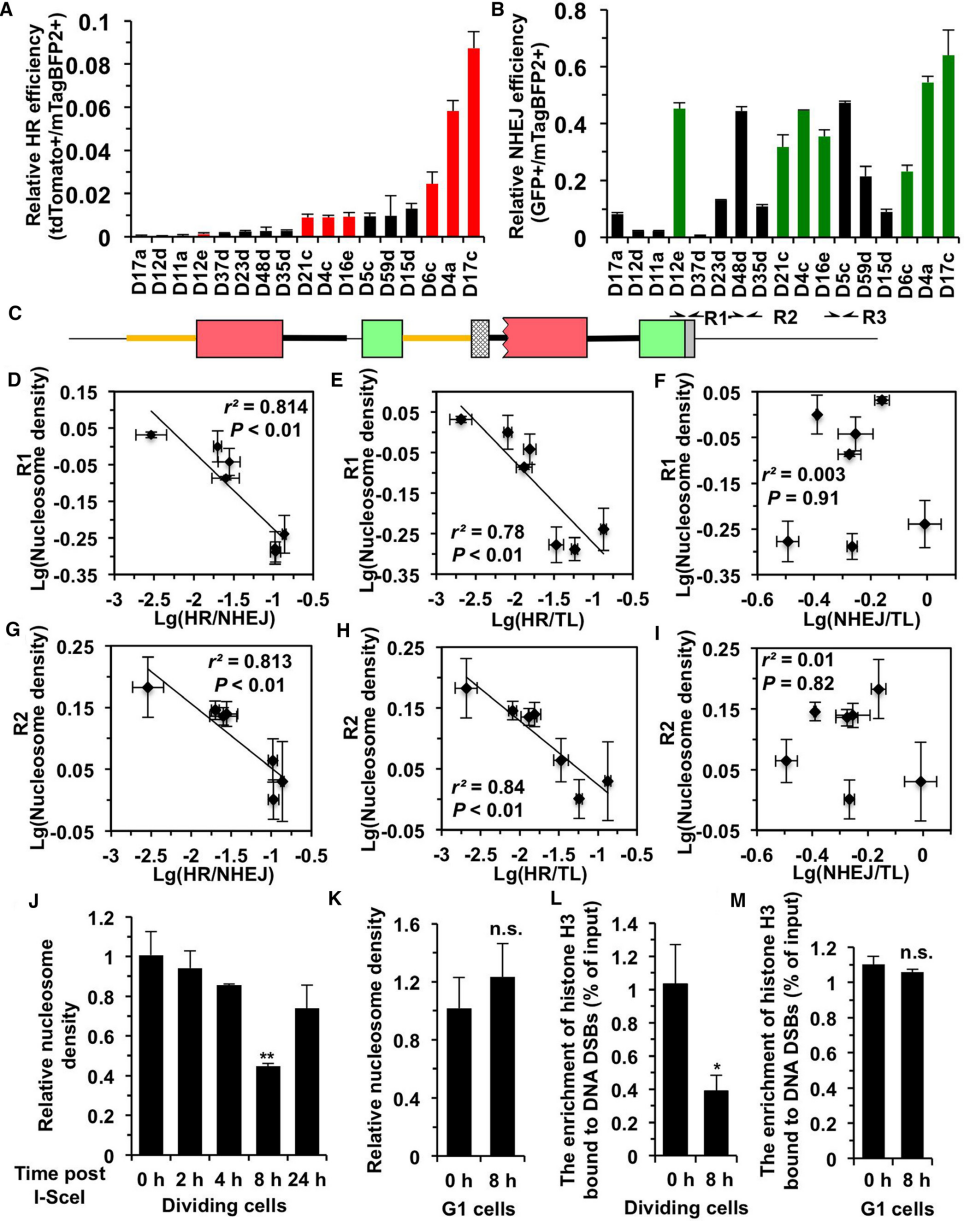
Nucleosome density negatively impacts DNA DSB repair by homologous recombination. HR (**A**) and NHEJ (**B**) repair efficiency was analyzed in 17 cell lines containing chromosomally integrated the HR-NHEJ reporter, described in Figure 1. Cell lines containing single copies of the HR-NHEJ reporter cassette were co-transfected with 2 μg I-SceI encoding plasmid and 0.005 μg pCMV-mTagBFP2 plasmid. On day 4 post transfection, cells were harvested for FACS analysis. The ratio of tdTomato+/mTagBFP2+ cells was used as a measure of HR efficiency (A). The ratio of GFP+/mTagBFP2+ cells was used as a measure of NHEJ efficiency (B). The 7 cell lines highlighted in color red or green have similar transcriptional level, cell cycle distribution and I-SceI cutting efficiency (Supplementary Figure S2A–C). (**C**) Schematic representation of the positions on the reporter for analysis of nucleosome density. Positions of R1-R3 are 2.8, 4, and 6-kb away from the I-SceI recognition sites, respectively. (**D–I**) The correlations between the choice of HR over NHEJ (HR/NHEJ: tdTomato+/GFP+), HR efficiency normalized to transcriptional level (tdTomato+/mTagBFP2+/relative transcription level), NHEJ efficiency normalized to transcriptional level (GFP+/mTagBFP2+/relative transcription level) and the densities of pre-existing nucleosomes at DNA damage sites (R1 and R2 regions). (**J**, **K**) Change of nucleosome density at DNA damage sites at different time points post I-SceI digestion in exponentially growing (J) or confluent (K) D4a cells. (**L**, **M**) Change of histone H3 enrichment at DNA damage sites in the presence or absence of I-SceI digestion in exponentially growing (L) or confluent (M) D4a cells. All experiments were repeated at least three times. Error bars represent s.d. ** *P* < 0.01

To study whether the nucleosome density affects the efficiency and the choice of the two DNA DSB repair pathways, we then examined the relative efficiency of HR and NHEJ across all 17 reporter-integrated cell lines. The repair efficiency for HR and NHEJ varied 256 and 86-fold between different cell lines (Figure 2A and B). This finding is consistent with reports that the efficiency of both HR and NHEJ is dependent on the location of the DSB in the genome (8,45). However, the observed efficiency of HR or NHEJ could be affected by a variety of parameters including the transcription level of CMV promoter at the specific integrated locus, the cell cycle distribution of the cell line and the cutting efficiency of I-SceI. We first examined the relative transcriptional level of the CMV promoter in the 17 cell lines by q-PCR, we found that indeed the transcription controlled by CMV promoter was position dependent (Supplementary Figure S3A). We then analyzed only nine cell lines with relatively high transcription level (at least 50% of the D4a cell line) (Supplementary Figure S3A). We further examined the cell cycle distribution of the 9 cell lines and found 2 of them had slightly higher number of cells in G1 (Supplementary Figure S3B). Then we quantified the I-SceI cutting efficiency by q-PCR in the seven remaining cell lines and we observed no significant difference between these cell lines (Supplementary Figure S3C).

Intriguingly in the seven cell lines with comparable transcription level, cell cycle distribution and cutting efficiency, the largest difference of HR efficiency was 72.6 fold while for NHEJ it was only 2.8 fold. We reasoned that variation in chromatin density across genomic loci may be modulating the differences in the choice of the two pathways and the DSB repair efficiency, and therefore set out to examine if the pre-existing nucleosomes affect the choice, and the efficiency of HR and NHEJ. According to the previously reported assay (16,35), we first calculated the relative density of pre-existing nucleosomes. Nuclei were isolated and digested with MNase, followed by DNA extraction and qPCR to determine the relative nucleosome density using primers at a 2.8 (R1), 4 (R2) and 6-kb (R3) distance to the I-SceI digestion site (Figure 2C). Correlation analysis revealed that nucleosome density at R1 or R2 regions strongly inversely correlated with the choice of HR over NHEJ (nucleosome density (ND) versus tdTomato+/GFP+) (R1: *r*^2^ = 0.814, *P <* 0.01) (R2: *r*^2^ = 0.813, *P <* 0.01) (Figure 2D and G). We then analyzed the correlation between nucleosome density and DNA repair efficiency. We found that either HR efficiency normalized to transcription level (tdTomato+/mTagBFP2+/Transcription level) or HR efficiency (tdTomato+/mTagBFP2+) strongly negatively correlated with nucleosome density at R1 (ND versus HR/TL: *r*^2^ = 0.78, *P* < 0.01) (ND versus HR: *r*^2^ = 0.66, *P* < 0.05) or R2 (ND versus HR/TL: *r*^2^ = 0.84, *P* < 0.01) (ND versus HR: *r*^2^ = 0.71, *P <* 0.05) regions (Figure 2E, H, Supplementary Figure S4A and C) while for NHEJ no significant correlation between the two variables was observed (Figure 2F, I, Supplementary Figure S4B, D). In contrast, at a distance of 6-Kb, the correlations were no longer significant (Supplementary Figure S4E–I).

Furthermore, we examined the kinetics of changes in nucleosome density in response to DNA double strand breaks using the D4a cell line. We found that at 8 h post I-SceI transfection in actively dividing cells, when HR repair occurred, the nucleosome density at R1 region declined by 50% (Figure 2J), while in confluent cells which were under G1 arrest and had nearly absent HR (Figure 1C) (46), the nucleosome density did not change after I-SceI transfection (Figure 2K). Moreover, we observed a similar reduction in nucleosome density at R2 locus which is 4-kb away from the I-SceI digestion (Supplementary Figure S4J). In contrast, we failed to observe any reduction at 6-kb (R3) or further regions (R4-7 kb and R5-8 kb) away from the I-SceI induced DNA DSBs (Supplementary Figure S4J). Here, we also validated the q-PCR assay for analyzing nucleosome density with ChIP assay using an antibody against histone H3. In line with the previous findings, we observed that the nucleosome occupancy at R1 region declined upon the occurrence of DNA DSBs in actively dividing cells while in G1 cells the reduction was abolished (Figure 2L, M). Cumulatively, these results suggest that the HR repair is sensitive to nucleosome density and that shedding nucleosomes is a critical step in the HR repair pathway.

### PARP1 is required for DNA DSB repair by HR through modulating nucleosome density at DNA damage sites

PARP1 is an NAD+ dependent enzyme that catalyzes the polymerization of ADP-ribose units on itself and other target proteins. It is chromatin associated and has been suggested to be involved in biological processes requiring relaxing chromatin structures such as transcription and DNA repair including NER and NHEJ (28), but whether and how PARP1 similarly regulates nucleosome density to facilitate the process of HR remains largely unknown.

**Figure 3.**
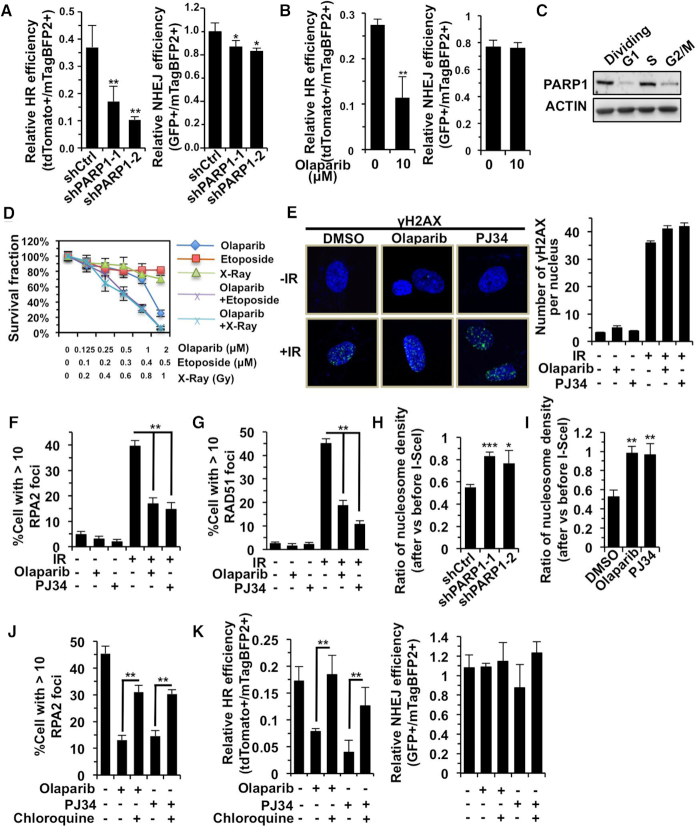
PARP1 regulates HR by modulating nucleosome density at DNA damage sites. (**A**) Depleting PARP1 has a more severe effect on HR than on NHEJ repair. (**B**) Inhibiting PARP1 by olaparib suppresses HR but not NHEJ. (**C**) Expression of PARP1 in different cell cycle stages. Cells were arrested in G1, S or G2 phase by confluency, aphidicolin (1 μg/ml) or colchicine (0.1 mg/ml) treatment respectively as previously reported (8). (**D**) Inhibiting PARP1 by olaparib sensitizes cells to DNA DSBs induced by either X-Ray or etoposide. (**E**) γH2AX foci formation at radiation-induced DSBs is not affected by PARP1 inhibition with olaparib or PJ34. (**F**) Quantification of RPA2 recruitment to radiation-induced DSBs upon PARP1 inhibitor olaparib or PJ34 treatment. At least 50 Geminin positive cells were counted on the fluorescence microscope, and only cells with over 10 RPA2 foci were counted as RPA2 foci positive. (**G**) Quantification of RAD51 recruitment to radiation-induced DSBs upon PARP1 inhibitor olaparib or PJ34 treatment. At least 50 Geminin positive cells were counted on the fluorescence microscope, and only cells with over 10 RAD51 foci were counted as RAD51 foci positive. (**H**) The ratio of relative nucleosome density at 2.8 kb away from break sites at 8 h post I-SceI transfections vs before I-SceI transfections in PARP1 depleted cells. (**I**) The ratio of relative nucleosome density at 2.8 kb away from break sites at 8 h post I-SceI transfections vs before I-SceI transfections in olaparib or PJ34 treated cells. (**J**) Pretreatment with chloroquine rescues the impaired recruitment of RPA2 to DNA damage sites in olaparib or PJ34 treated HCA2-hTERT cells. At least 50 Geminin positive cells were counted on the fluorescence microscope, and only cells with over 10 RPA2 foci were counted as RPA2 foci positive. (**K**) Pretreatment with chloroquine rescues the decline of HR in olaparib or PJ34 treated D4a cells. All experiments were repeated at least three times. Error bars represent s.d. * *P* < 0.05, ** *P* < 0.01, *** *P* < 0.001.

We therefore set out to confirm that PARP1 participates in DNA DSB repair. We found that both depleting PARP1 using shRNA and inhibiting PARP1 enzymatic activity to suppress the formation of poly ADP ribose (PAR) using olaparib and PJ34, two PARP inhibitors, had strong inhibitory effects on HR, while for NHEJ the suppressive effect was relatively mild (Figure 3A, B and Supplementary Figure S5A–C). We also confirmed the inhibitory effect of depleting PARP1 or inhibiting PARP1 enzymatic activity in a well-established HCA2-H15c cell line, which is a clone of the HCA2-hTERT cell harboring a single copy of HR reporter cassette (Supplementary Figure S5D–G) (8,36,46). Since HR is predominantly utilized in S phase when sister chromatids are available for homology search, we examined the expression level of PARP1 in different cell cycle stages. We found that PARP1 expression reached its peak in S phase, and more PAR chains were synthesized upon X-Ray treatment than those in G1 arrested cells, suggesting that PARP1 is probably a critical factor in HR (Figure 3C, and Supplementary Figure S5H). More importantly, we demonstrated that blocking PARP1 enzymatic activity using olaparib sensitized HCA2 cells to DNA DSBs induced either by X-Ray or etoposide (Figure 3D), indicating that PARP1 is a critical factor for repairing DNA DSBs. Intriguingly, since HR is often up-regulated in cancerous cells (47,48), one would expect that blocking PARP1 with olaparib would sensitize cancer cells rather than normal cells to chemicals inducing DNA DSBs. Indeed, we found that treating cancer cell lines including MCF7, Hep3B and HeLa with olaparib greatly enhanced the sensitivity of cells to etoposide (Supplementary Figure S6). In contrast, in normal cell lines such as MCF10A and Changliver with low HR capacity, no synergistic effect could be observed (Supplementary Figure S6). Taken together, these results indicate that combining PARP1 inhibitors and chemicals inducing DNA DSBs holds the potential to treat HR proficient cancers.

To identify the step at which PARP1 regulates DNA DSB repair, we performed immunostaining experiments. Inhibiting PARP1 enzymatic activity with either olaparib or PJ34 did not influence the γH2AX formation (Figure 3E), while inhibiting PARP1 affected the recruitment of both RPA2 and RAD51, which play roles in end resection and recombination (Figure 3F–G, Supplementary Figure S7A, B), which confirms a recent finding that demonstrated defects in RAD51 loading at damaged sites upon PARP inhibitor treatment (49). Furthermore, cell cycle analysis demonstrated that neither olaparib nor PJ34 reduced the proportion of cells in S/G2 stage, in which HR occurs (Supplementary Figure S7C). Even at a low concentration, which did not cause any obvious cell cycle alteration, HR was still significantly inhibited (Supplementary Figure S7D). In addition, we found that RPA2 did not interact with either PAR or PARP1 in the absence or presence of DNA damage (Supplementary Figure S8A, B). These pieces of data strongly suggest that during the process of HR, PARP1 participates in the step between the DNA damage response and end resection.

We therefore examined the change of nucleosome density at DNA damage sites in cells transduced with PARP1 shRNA or treated with PARP1 inhibitors. We found that both methods abrogated the reduction of nucleosome density at DNA DSB sites (Figure 3H-I, Supplementary Figure S8C-D). More intriguingly, by forcing the relaxation of chromatin using chloroquine (16), the recruitment of RPA2 to DNA damage sites was significantly stimulated in olaparib or PJ34 treated cells (Figure 3J, Supplementary Figure S8E). Pretreatment with chloroquine or valproic acid (VPA), a HDAC inhibitor, led to significantly improved HR efficiency in cells treated with PARP1 inhibitors (Figure 3K, Supplementary Figure S8F). Taken together, these data indicate that PARP1 regulates HR by reducing nucleosome density at DNA damage sites.

Since both olaparib and PJ34 act as PARP1 inhibitors by abrogating its enzymatic activity (50,51) and had no effect on its protein level (Supplementary Figure S8G), and both depleting PARP1 and inhibiting PARP1 led to reduced HR and impaired chromatin relaxation in response to DNA damage, we hypothesized that the PAR mediated recruitment of chromatin remodelers to DNA damage sites is responsible for clearing nucleosomes at broken DNA ends, therefore facilitating HR.

### BRG1 recruited by PAR reduces nucleosome density to promote HR at DNA DSB sites

**Figure 4.**
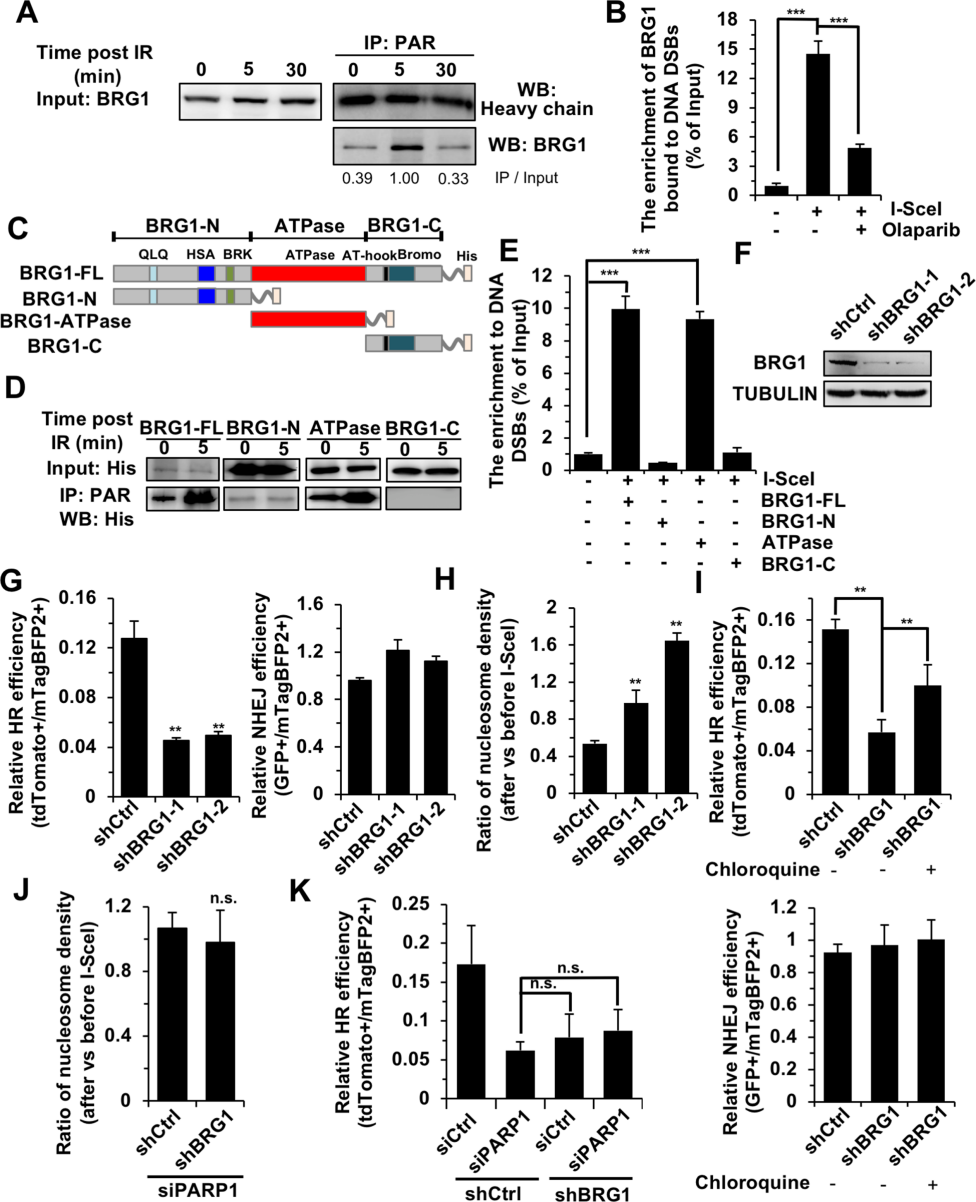
BRG1 recruited by PAR regulates chromatin relaxation to facilitate HR. (**A**) Co-IP and WB analysis of the interaction between BRG1 and PAR in response to IR. (**B**) ChIP analysis of BRG1 enrichment at DNA damage sites induced by I-SceI digestion in the presence or absence of the PARP1 inhibitor, olaparib. (**C**) Schematic representation of the BRG1 domain structure, and the truncated constructs used in this study. (**D**) Co-IP and WB analysis of the interaction between PAR and the three domains of BRG1 in the presence or absence of DNA DSBs induced by X-Ray. (**E**) Analysis of recruitment of three BRG1 domains to DSBs using ChIP assay. Different amounts of GFP tagged full length BRG1 and three BRG1 domains were transfected into NHEJ-I9A cells to ensure equal expression of them. Then ChIP was carried out by using an antibody against GFP. **(F)** WB analysis of D4a cells with BRG1 depleted using two shRNAs against BRG1 integrated into the genome. (**G**) Depleting BRG1 significantly impairs DNA DSB repair by HR, but not NHEJ. (**H**) The ratio of nucleosome density at 2.8 kb away from break sites at 8 h post I-SceI transfections vs before I-SceI transfections with or without BRG1 depletion. (**I**) Pretreatment with chloroquine rescues the decline of HR, but not NHEJ, in BRG1 depleted cells. (**J**) Epistasis analysis of PARP1 and BRG1 effect on nucleosome density. (**K**) Epistasis analysis of PARP1 and BRG1 effect on HR repair. All experiments were repeated at least three times. Error bars represent s.d. ** *P* < 0.01, *** *P* < 0.001, n.s. not significant.

To test the hypothesis that PAR mediates the recruitment of a chromatin remodeler to DNA DSB sites, we performed mass spectrometry on samples immunoprecipitated with an antibody against PAR in the absence and presence of IR. The analysis of mass spectrometry results indicates that upon DNA damage PAR interacted with BRG1, a critical ATPase dependent chromatin remodeler (Supplementary Figure S9A). BRG1 has been reported to be involved in DNA DSB repair independent of its ATPase activity (19,24), but whether BRG1 participates in HR by clearing nucleosomes at DNA DSB sites in an ATPase dependent manner has not been characterized. We first confirmed the interaction between PAR and BRG1 using co-IP and Western blot (Figure 4A). We also performed an *in vitro* experiment by incubating purified recombinant BRG1-His and biotin labeled PAR, followed by streptavidin pull-down and western blot analysis using an antibody against His. The result indicates that PAR directly interacted with BRG1 *in vitro* (Supplementary Figure S9B). In addition, we found that BRG1 was not parylated upon DNA damage (Supplementary Figure S9C).

Our ChIP assay using NHEJ-I9a reporter cells demonstrated that BRG1 was recruited to broken ends and this recruitment was abolished in the presence of the PARP1 inhibitors, olaparib (Figure 4B). Further experiments revealed that ATPase domain rather than N- or C- terminal domains of BRG1 interacted with PAR in response to DNA damage (Figure 4C, D). *In vitro* biochemical reactions confirmed that BRG1 ATPase domain interacted with PAR (Supplementary Figure S9D). Additionally, the BRG1 ATPase domain but not the N- or C- terminal domains may successfully be recruited to DNA damage sites (Figure 4E). These data indicated that similar to several other members of BAF complex, BRG1 is recruited to DNA DSB sites in a PARP1-dependent manner (52)_._

To understand the function of BRG1 at DNA damage sites, we depleted BRG1 in D4a and HCA2-H15c cells (Figure 4F) and analyzed DNA DSB repair efficiency. Our results suggest that HR but not NHEJ was significantly reduced (Figure 4G, Supplementary Figure S10A, B). BRG1 depletion did not affect cell cycle distribution (Supplementary Figure S10C). We also failed to observe the reduction of nucleosome density following the induction of a DNA DSB in BRG1-depleted cells (Figure 4H, Supplementary Figure S10D). In addition, the suppression of HR was also significantly rescued by chloroquine or VPA, suggesting that the regulation of HR by BRG1 is dependent on its chromatin remodeling activity (Figure 4I, Supplementary Figure S10E). We then analyzed the HR efficiency and the change of nucleosome density in both PARP1 and BRG1 depleted cells. No significant difference in HR efficiency and nucleosome density was observed between depleting both genes and either gene singly (Figure 4J-K, Supplementary Figure S10F), demonstrating that the two proteins are in the same repair pathway. However, we did not observe *in vitro* nucleosome remodeling activity by BRG1 in the presence of PARP1 (Supplementary Figure S10G), suggesting that an additional factor might be needed for the function of BRG1 at DNA damage sites.

### PAR-mediated recruitment of SIRT1 is required for clearing nucleosomes and promoting HR at DNA DSB sites

**Figure 5.**
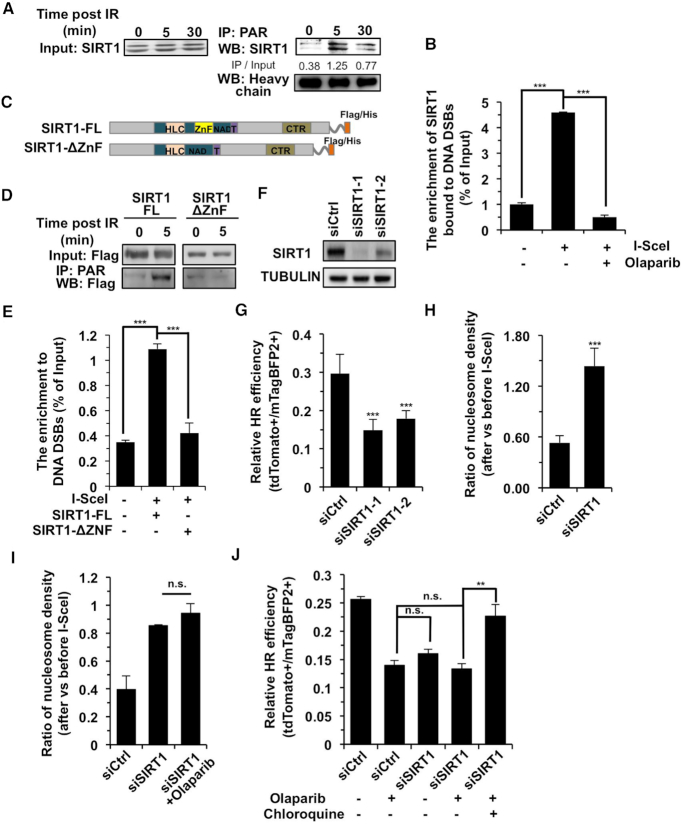
SIRT1 recruited by PAR is required for nucleosome clearance at DNA DSB sites and HR directed repair. (**A**) SIRT1 interacts with PAR in response to IR. (**B**) ChIP analysis of SIRT1 enrichment at DNA damage sites induced by I-SceI digestion in the presence or absence of the PARP1 inhibitor, olaparib. (**C**) Schematic representation of the Flag tagged full length and ZnF deleted SIRT1 (ΔZnF). (**D**) Only full length SIRT1 interacts with PAR in response to IR. The interaction was tested using co-IP and WB analysis. (**E**) ChIP assay demonstrated that SIRT1 (ΔZnF) failed to be recruited to DSBs. (**F**) Endogenous SIRT1 is depleted using siRNA transfection. (**G**) SIRT1 depletion causes reduced HR efficiency. (**H**) The ratio of nucleosome density at 2.8 kb away from break sites at 8 h post I-SceI transfections vs before I-SceI transfections with or without SIRT1 depletion. (**I**) Epistasis analysis of PARP1 and SIRT1 effect on nucleosome density. (**J**) Epistasis analysis of PARP1 and SIRT1 effect on HR repair. Pretreatment with chloroquine rescues the reduction in HR efficiency in D4a cells with PARP1 inhibited by olaparib and SIRT1 depleted. All experiments were repeated at least three times. Error bars represent s.d. ** *P* < 0.01, *** *P* < 0.001, n.s. not significant.

Further analysis of our mass spectrometry data revealed that SIRT1, which is a deacetylase involved in DNA repair particularly by HR (53,54), also interacted with PAR upon DNA damage (Supplementary Figure S9A). We then performed co-IP and WB experiments. Our results confirmed that SIRT1 interacted with PAR in response to the induction of DNA DSBs (Figure 5A). Furthermore, ChIP assay using NHEJ-I9a reporter cells demonstrated that the recruitment of SIRT1 to DNA damage sites was dependent on PAR (Figure 5B).

PAR has been reported to interact with the ZnF domain (55), which is also found in SIRT1. We therefore hypothesize that the ZnF domain mediates the recruitment of SIRT1 to DNA DSB sites by interacting with PAR. We created a vector expressing Flag or His tagged SIRT1 with deleted ZnF domain (Figure 5C). We found that in response to DNA damage loss of ZnF domain abrogated the interaction between SIRT1 and PAR (Figure 5D). We also found that the interaction between PAR and SIRT1 was abolished in the absence of the ZnF domain *in vitro* (Supplementary Figure S11A). In addition, SIRT1-ΔZnF failed to be recruited to DNA DSB sites (Figure 5E). Moreover, depleting SIRT1 affected both HR efficiency and nucleosome density change at DNA damage sites (Figure 5F–H, Supplementary Figure S11B–D), indicating that SIRT1 regulates HR through chromatin relaxation.

To confirm that PAR and SIRT1 function in the same pathway of regulating HR directed repair, we examined the nucleosome density change and HR efficiency in SIRT1 depleted cells pretreated with olaparib or PJ34. We found that blocking PARP1 activity did not cause additional changes in nucleosome density or further reduction of HR in SIRT1 depleted cells (Figure 5I, J, Supplementary Figure S11E–G). In addition, supplementation with chloroquine significantly rescued the declined HR (Figure 5J, Supplementary Figure S11H), strongly indicating that SIRT1 regulates HR by promoting the relaxation of chromatin at DNA damage sites.

### SIRT1 directly deacetylates BRG1 in response to DNA damage

**Figure 6.**
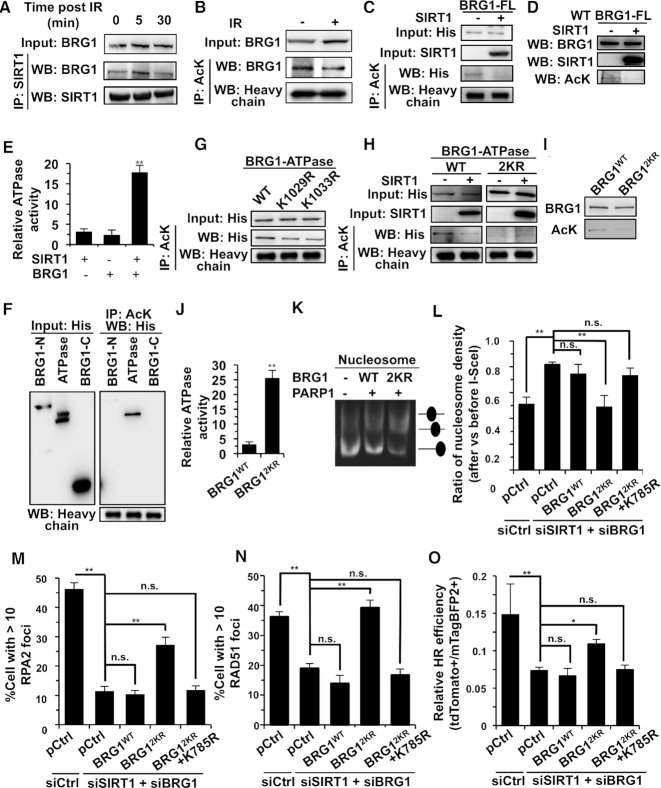
In response to IR, SIRT1 deacetylates BRG1 to promote its ATPase activity, thereby stimulating nucleosome sliding to facilitate HR. (**A**) Analysis of the interaction between SIRT1 and BRG1 in response to IR. (**B**) Analysis of BRG1 acetylation levels in response to IR. (**C**) Co-IP analysis of BRG1 acetylation in 293T cells overexpressing SIRT1. (**D**) BRG1 is deacetylated by SIRT1 *in vitro*. The recombinant BRG1 was purified from 293F cells and incubated with recombinant SIRT1 for the deacetylation reaction. (**E**) BRG1 deacetylated by SIRT1 has higher ATPase activity than BRG1 with no SIRT1 treatment. The recombinant BRG1 was purified from 293F cells and incubated with recombinant SIRT1 before being subjected to the analysis of ATPase activity using the malachite green ATPase assay. (**F**) Co-IP analysis of acetylation levels of the three domains of BRG1. His-tagged three domains of BRG1 were transfected to 293F cells before co-IP and western blot analysis was performed. (**G**) Acetylation level of BRG1 ATPase domain WT and two mutants, K1029R and K1033R. His-tagged WT or mutated ATPase domain of BRG1 was transfected to 293T cells before co-IP and western blot analysis was performed. (**H**) Co-IP analysis of acetylation level of WT and the 2KR mutant containing both K1029R and K1033R mutations of BRG1-ATPase domain in 293T cells overexpressing SIRT1. (**I**) Acetylation level of purified recombinant BRG1 WT and the 2KR mutant. (**J**) Analysis of ATPase activity of BRG1 WT and 2KR mutant using the malachite green ATPase assay. (**K**) Analysis of nucleosome sliding activity of BRG1 WT and 2KR mutant in the presence of PARP1. (**L**) In both SIRT1 and BRG1 depleted cells only overexpressing BRG1 2KR mutant but not the WT or the ATPase dead mutant K785R rescues the impaired nucleosome clearance at DNA damage sites. (**M, N**) Quantification of RPA2 and RAD51 foci positive cells upon induction of DNA DSBs in both SIRT1 and BRG1 depleted cells. Cells transfected with different siRNA and expression vectors were irradiated on an X-Ray machine. At 4 h post IR, cells were fixed for immunofluorescence experiments. At least 50 Geminin positive cells were counted and only cells with over 10 RPA2 or RAD51 foci were counted as foci positive. In both SIRT1 and BRG1 depleting cells only overexpressing BRG1 2KR mutant but not the WT or the ATPase dead mutant K785R can rescue the impaired recruitment of RPA2 and RAD51 to DNA damage sites. (**O**) In SIRT1+ BRG1 depleted cells only overexpressing BRG1 2KR mutant, but not the WT BRG1 or the ATPase dead mutant K785R, partially rescues the reduced HR efficiency. All experiments were repeated at least three times. Error bars represent s.d. * *P* < 0.05, ** *P* < 0.01, n.s. not significant.

Since both BRG1 and SIRT1 are involved in chromatin relaxation thereby promoting DNA DSB repair, we speculated that there is a crosstalk between the two factors. We tested if SIRT1 interacts with BRG1 using co-IP. We found that SIRT1 interacted with BRG1 in response to DNA damage (Figure 6A). In addition, we found that the acetylation level of BRG1 was reduced upon IR (Figure 6B). Since SIRT1 is a deacetylase, we further tested if BRG1 was deacetylated by SIRT1. By performing co-IP analysis we found that overexpression of SIRT1 led to a reduced acetylation level of BRG1 *in vivo* (Figure 6C). To further demonstrate that the deacetylation of BRG1 by SIRT1 is a direct effect, we purified both BRG1 and SIRT1 in 293F cells and then performed *in vitro* deacetylation assay. We observed that SIRT1 deacetylated BRG1 *in vitro* (Figure 6D), indicating that BRG1 is a direct target of SIRT1.

### SIRT1 deacetylates BRG1 at residues K1029 and K1033 to promote its ATPase activity and facilitate chromatin relaxation to stimulate HR

To understand the biological function of BRG1 deacetylation, we first performed *in vitro* analysis of ATPase activity using malachite green assay. We found that *in vitro* deacetylation of BRG1 by SIRT1 increased its ATPase activity by ∼7.4-fold (Figure 6E). We then set out to uncover which sites of BRG1 are deacetylated by SIRT1. We examined the acetylation level of the N-, C- and ATPase domains of BRG1 in mammalian cells using co-IP assay. We found that the ATPase domain, rather than the other two domains, was acetylated (Figure 6F). *In vitro* biochemical deacetylation reactions using purified recombinant BRG1-ATPase and SIRT1 confirmed that SIRT1 deacetylated the BRG1 ATPase domain directly (Supplementary Figure S12A). To identify potential deacetylation sites of BRG1 by SIRT1, we predicted potential deacetylated sites by SIRT1 as previously reported (Supplementary Figure S12B) (56). We then introduced K→R mutations in the BRG1 ATPase domain and performed co-IP experiments. We found that among all the potential lysine sites, K1029R and K1033R mutations but not other mutations reduced the BRG1 acetylation level (Figure 6G, Supplementary Figure S12C). We then overexpressed SIRT1 and examined the acetylation level of BRG1 WT, 2KR (K1029R and K1033R) mutant. We observed a strong reduction of acetylation level in BRG1 WT (Figure 6H), while the decreased acetylation level of BRG1 2KR was not further reduced in the presence of SIRT1, confirming that the two sites are deacetylated by SIRT1.

To further examine the functional difference between WT and the 2KR mutant with both sites mutated to arginine, we purified recombinant full length BRG1-WT and BRG1-2KR and compared the acetylation level of the two proteins. We found that the 2KR mutant was less acetylated than WT (Figure 6I). We then assessed the ATPase activity of the two recombinant proteins. We found that the ATPase activity of the BRG1 2KR mutant was ∼ 10-fold higher than that of BRG1 WT *in vitro* (Figure 6J), which was consistent with the high ATPase activity of the deacetylated BRG1 (Figure 6E). To further understand whether the 2KR mutant facilitates the nucleosome sliding, we performed *in vitro* nucleosome sliding experiments as previously described (31,39). We demonstrated that the BRG1 2KR mutant was more active at sliding of nucleosomes in comparison to BRG1 WT (Figure 6K).

To further confirm that the BRG1 2KR mutant promotes HR by clearing nucleosomes at DNA DSB sites *in vivo*, we then analyzed whether the restoration of 2KR mutant could rescue the clearance of nucleosomes at DNA lesions, recruitment of RPA2 and RAD51 to damage sites, and HR efficiency in D4a cells with both endogenous BRG1 and SIRT1 depleted. Indeed, our experiment demonstrated that overexressing 2KR mutant but not WT could rescue the clearance of nucleosomes at DNA damage sites in both BRG1 and SIRT1 depleted cells (Figure 6L). The immunofluorescence experiments also indicated that expression of the 2KR mutant rescued the decline of RPA2 and RAD51 recruitment to DNA damage sites (Figure 6M, N, Supplementary Figure S13A, B). Moreover, we also observed that the 2KR mutant but not the WT partially rescued the reduced HR efficiency in the absence of both SIRT1 and BRG1 (Figure 6O). Intriguingly, inactivating the ATPase activity by introducing K785R mutation on the 2KR mutant abrogated the effects of rescuing nucleosome clearance, RPA2 and RAD51 recruitment and HR repair (57) (Figure 6L-O, Supplementary Figure S13A-B), strongly confirming that BRG1 regulates chromatin relaxation and HR repair in an ATPase dependent manner. Taken together, we demonstrate that the deacetylated BRG1 activates ATPase activity, clears the nucleosomes, facilitates the recruitment of RPA2 and RAD51, and promotes HR directed repair.

## DISCUSSION

We generated a novel HR-NHEJ reporter cassette and a series of cell lines harboring this cassette at different genomic loci. Using these tools, we delineated an axis leading to nucleosome remodeling at the DNA DSB site, where PARP1 generates PAR molecules as an early response to DNA DSBs, the PAR then recruits SIRT1 and BRG1; SIRT1 deacetylates BRG1 on the residues K1029 and K1033 stimulating the removal of nucleosomes by BRG1, therefore promoting HR directed repair (Supplementary Figure S15). This work demonstrates the function of PARP1 in DNA DSB repair, laying the foundation for applying PARP1 inhibitors to cancer treatment by inhibiting the hyper-activated HR repair machinery in tumors.

### The advantages of the novel HR-NHEJ reporter cassette

Numerous fluorescent reporter substrates for the analysis of DNA DSB repair have been developed (9,34,41,58,59), providing spectacular advances in the DNA repair field. In comparison to previously published substrates, our cassette is efficient at measuring both HR and NHEJ repair outcomes at the same break site. The classical way of analyzing both HR and NHEJ repair products was recovering the repair products through the use of antibiotic selection and analyzing them by Southern blot (60). This approach, however, is labor intensive and can only analyze a small number of events. Two systems had been reported that could measure both HR and NHEJ. In one system, two separate reporter constructs based on different fluorescent genes were integrated into the genome of one cell line to simultaneously measure HR and NHEJ at different loci (61). This system is a good attempt to study the dynamic shifts between HR and NHEJ, nevertheless, it does not allow for accurate comparisons between the two repair processes since HR and NHEJ are strongly influenced by chromosomal positions. The second system, named ‘traffic light’ measures HR and NHEJ repair outcomes at the same chromosomal locus using an exogenous donor template. This system is ideal for studies of genome engineering but does not recapitulate endogenous repair (45). Furthermore, the NHEJ repair cassette in this construct detects only a fraction of repair events, limited to small deletions associated with 2 bp frameshifts (45), limiting the utility of this construct for driving insight into the biology of DNA DSB repair.

The key advantage of our dual reporter is that it can detect a wide spectrum of NHEJ repair events with exception of very large deletions spanning beyond the Pem1 intron and tdTomato ORF (∼4 kb). Furthermore, our dual reporter does not require an exogenous template since it contains a large ∼3.9 kb region of homology for HR repair, that is more reminiscent of the interchromatid HR than constructs with short homology regions. Furthermore, our reporter detects two major non-mutagenic HR outcomes, gene conversion and crossing-over. Finally, HR and NHEJ repair outcomes are measured at the same break site, accurately reflecting the interplay between the two repair processes.

### The novel role of PARP1 in DNA DSB repair

Roles for PARP1 in other types of DNA repair, including BER, SSB repair, and NER, have been well defined (28). Recent reports have extended the function of PARP1 to both c-NHEJ and alt-NHEJ (20,30). Similar to NHEJ, roles of PARP1 in HR have received great attention. Independent of its enzymatic activities, PARP1 interacts with Timeless at DNA DSB sites to promote HR, possibly stabilizing collapsed DNA replication forks (62). Additionally, upon DNA damage, PARP1 catalyzes the synthesis of negatively charged poly-ADP-ribose chains on itself, and thereby recruits several important DNA damage responsive factors with BRCT and FHA domains to DNA DSB sites to initiate the HR signaling cascade (28). However, there are some debates on the role of PARP1 in HR repair. A previous study indicates that PARP1 knock-down or inhibition had no effect on HR repair (63), while another study reported that PARP1 knock-down impaired HR (62), both using the U2OS-DR-GFP cell line. The discrepancies between previous reports and our work might result from a lot of factors, such as the type of cell lines, the genomic locus where the reporter was integrated, knocking down efficiencies, or the type/dosage of inhibitors. Besides confirming PARP1 deficiency impairing HR, using both interfering RNA and PARPi, our findings further clarify the mechanisms by which PARP1 promotes HR—namely, by recruiting BRG1 and SIRT1 to DNA DSB sites to promote chromatin relaxation between the steps of signaling and end resection. Whether PARP1 is involved in rate limiting steps of HR such as the two steps of end resection, strand invasion, and homology search remains to be further determined.

Previous work has shown that knocking out essential HR factors in mice leads to embryonic lethality (64–66), while knocking out PARP1, which is required for HR based on our data, does not have such severe consequences. Our finding that PARP1 regulating nucleosome clearance at DNA DSB sites can probably reconcile these seemingly contradictory observations. During embryogenesis, rapidly proliferating cells undergo strong replication stress, which requires HR to relieve, therefore HR is an extremely important pathway to safeguard the genomes during the process. However, in comparison to that, in terminally differentiated cells, the chromatin in embryonic stem cells is probably less compact (67). Therefore, upon the induction of DNA DSBs in ES cells, the PAR-dependent nucleosome clearance at DNA lesions is probably not a necessary step. Nevertheless, further experiments are needed to investigate the roles of PARP1 in DNA DSB repair in ES cells and other types of stem cells.

The concept of applying PARP1 inhibitors to treat HR deficient cancers, such as breast cancers with familial BRCA1 or BRCA2 mutations, has largely been based on the assumption that drugs targeting PARP1 impair BER or SSB repair, therefore leading to the generation of massive DSBs in cells which cannot be fixed by defective HR repair machinery, eventually causing cell death by activating p53—a form of synthetic lethality. Our results describing the novel function of PARP1 in HR repair suggest that inhibiting PARP1 might be a good target of treating tumors with hyperactive HR pathways. Indeed, several PARP1 inhibitors in clinical trials and approved by governmental agencies are not limited to familial breast cancers (68). They have been extended to non-small-cell lung cancer, prostate cancer, colorectal cancer, and others, strongly suggesting that PARP1 may have additional roles beyond repairing damaged bases or SSBs. Particularly, one report indicates the efficacy of rucaparib, a PARP1 inhibitor, is positively correlated with HR capacity in HR competent ovarian cancer (69). Nevertheless, whether PARP1 inhibitors function to treat cancer by inhibiting HR needs to be tested in knock-in mice models for assessing HR efficiency *in vivo*.

### The ATPase activity of BRG1 is critical in HR directed repair

The ATPase dependent chromatin remodeler BRG1 is the core subunit of the SWI/SNF complex. BRG1 is currently well characterized as a tumor suppressor. Mutations in BRG1 are frequently observed in lung cancer cell lines and several types of primary tumors (70). In addition, BRG1 heterozygous mice are prone to mammary tumorigenesis in comparison to WT (71). Our data mining analysis indicates that the mutation frequency on the ATPase domain is higher than other domains in both lung cancer cell lines and primary lung cancers (Supplementary Figure S14A-B) (72,73). Since it has been well documented that aberrant HR repair is associated with tumorigenesis (74), one would expect that if BRG1 takes part in HR, then mutations to the ATPase would play a pivotal role in tumorigenesis.

However, surprisingly, previous reports have suggested that independent of its ATPase activity, ATM mediated phosphorylation of BRG1 facilitates the expansion of γH2Ax upon DNA damage while BRG1 collaborates with RAD52 to expedite the process of loading RAD51 onto single stranded DNA before strand invasion and homology search (19,24). Intriguingly, our findings demonstrate that the SIRT1 mediated deacetylation of BRG1 occurs on the ATPase domain and the deacetylation greatly potentiates its enzymatic activity and ATPase-dependent chromatin remodeling activity, therefore sliding the nucleosomes away from DNA damage sites and promoting HR.

Nevertheless, BRG1 is also involved in other biological processes such as transcriptional activation or repression. For instance, a previous study indicates that in actively transcribed regions of chromatin BRG1 catalytic ATPase domain repressed transcription upon the induction of DNA damage (75), which seems contradictory to our report here. We reasoned that BRG1 is involved in the temporal and spatial regulation of DNA repair. At the steps of DNA damage response, BRG1 suppresses the transcription possibly by condensing chromatins surrounding the promoter region, while at the steps of DNA repair, BRG1 relaxes chromatins to promote HR at DSB sites. Furthermore, whether and how the modification of its ATPase domain, such as through deacetylation by SIRT1, affects the expression of critical genes involved in tumorigenesis remains to be further determined.

### Multiple roles of SIRT1 in DNA DSB repair by HR

SIRT1 is a chromatin-associated deacetylase. It targets many essential factors involved in cell survival, cell metabolism, chromatin regulation, transcription and DNA repair (76). Its role as a deacetylase in DNA DSB repair has also been studied. Upon DNA damage it is redistributed across chromosomes, and depleting SIRT1 impairs the recruitment of RAD51 to DNA damage sites (53). In addition, SIRT1 deacetylates NBS1, a member of the MRN complex, to promote ATM mediated NBS1 phosphorylation and to facilitate the process of HR. Moreover, the RecQ helicase, WRN, which binds specifically to Holliday junctions to promote their resolution at the late stage of HR (77), is also deacetylated and activated by SIRT1 (78). Our work adds another important piece of evidence showing that SIRT1 activates BRG1 to relax the chromatin architecture at DNA DSB sites to promote HR. These studies demonstrate that SIRT1 acts as a HR repair factor at multiple steps of DNA DSB repair. It is worth noting that previous work from our and others’ groups demonstrate that SIRT6, another member of Sirtuin family, promotes HR through activating PARP1 and facilitating the recruitment of SNF2H to broken ends (16,36). Although it would be interesting to further examine whether there is a direct crosstalk between SIRT1 and SIRT6 during the regulation of HR, our data here indicates that PARP1 is the central protein in the interplay between these two critical Sirtuin proteins.

## Supplementary Material

gkz592_Supplemental_Files
