# Supplementary material for: A PARP1–BRG1–SIRT1 axis promotes HR repair by reducing nucleosome density at DNA damage sites
Source: Nucleic Acids Res. 2019 Jul 10;47(16):8563–80. doi: 10.1093/nar/gkz592 (PMC7145522; doi:10.1093/nar/gkz592)
Supplement: gkz592_Supplemental_Files [file gkz592_supplemental_files.zip › Supplementary Figure Legends.pdf]

Supplementary Figure and Table Legends

Supplementary Figure 1. (A) Validation of HR and NHEJ repaired products. 5 μg of control vector pHPRT, pHR, pNHEJ were transfected into HCA2-hTERT cells. On Day 3 post transfection, cells were harvested for FACS analysis. **(B)** HCA2-hTERT cells containing the dual fluorescent reporter cassette were infected with Adenovirus encoding the I-SceI endonuclease (1). At day 5 post infection, pictures were taken on a Leica inverted fluorescent microscope. **(C)** The copy number of dual reporter cassettes in the genomes of HCA2-hTERT cell lines. Cells were cultured to complete confluence and harvested for genomic DNA extraction. The extracted DNA was used as a template for quantitative PCR to analyze the copy number of the reporter cassette. Comparative CT Method ($2^{-\Delta\Delta CT}$) was used to calculate the relative copy number. **(D-E)** Three fluorochromes encoded by pEGFP-N1, pDsRed2-N1 or pCMV-mTagBFP2 can be analyzed simultaneously with no interference. Separate aliquots of HCA2-hTERT cells were transfected with 5 μg each of pEGFP-N1, pDsRed2-N1 or pCMV-mTagBFP2 vectors. On day 3 post transfection, cells were harvested for FACS analysis. We previously showed that EGFP does not interfere with DsRed (2), therefore, here we analyzed the potential interference between EGFP and mTagBFP2 or between DsRed2/tdTomato (the two share the same excitation and emission wavelength) and mTagBFP2. Fluorescent proteins encoded by pEGFP-N1 and pCMV-mTagBFP2 did not show interference during detection (D). Fluorescent proteins encoded by pDsRed2-N1 and pCMV-mTagBFP2 did not show interference during detection (E).

Supplementary Figure 2. (A) Cell cycle distribution of D4a cells after the indicated treatment. Confluent cells were harvested for cell cycle analysis on Day 10 after these cells exhibited a phenotype of contact inhibition. Cells arrested in S phase were treated with aphidicolin for 4 days before being harvested for cell cycle analysis. **(B)** HR is strongly inhibited by MRE11 activity blockage using Mirin in cells containing chromosomally integrated HR-NHEJ reporter cassette. **(C-D)** Knocking down both DNA-PKcs and PARP1 (C) or DNA-PKcs and POLθ (D) dramatically inhibited NHEJ in cells containing chromosomally integrated HR-NHEJ reporter cassette. Cells were transfected with indicated siRNAs twice with a 2-day interval. Vectors encoding I-SceI and mTagBFP2 were then co-transfected to cells. On day 2 post transfection of I-SceI and mTagBFP2 vectors, cells were harvested for FACS analysis. The sequences of siRNAs against DNA-PKcs, PARP1 and POLθ are as follows: DNA-PKcs, 5'-GAUCGCACCUUACUCUGUUTT-3'; PARP1, 5'-CGACCUGAUCUGGAACAUCAATT-3'; POLθ, a pool of the following four siRNAs: 5'-CCUUAAGACUGUAGGUACUTT-3', 5'-ACACAGUAGGCGAGAGUAUTT-3', 5'-CGACUAAGAUAGAUCAUUUTT-3', 5'-ACAACAACCCU UAUCGUAAATT-3'. To measure the relative POLθ mRNA level, the total RNA extracted from siRNA transfected D4a cells was reverse transcribed into cDNA. Then the real-time PCR was performed with the cDNA and the comparative CT Method ($2^{-\Delta\Delta CT}$) was employed to quantify the relative POLθ mRNA level. The sequences of primers for amplifying POLθ and the control GAPDH are as follows: POLθ-Forward 5' CTACAAGTGAAGGGAGATGAGG 3', POLθ-Reverse, 5' TCAGAGGGTTTCACCAATCC 3'; GAPDH-Forward, 5' TGGTATGACAACGAATTTGG 3', GAPDH-Reverse, 5' TCTACATGGC AACTGTGAGG 3'. **(E)** NHEJ is strongly inhibited by DNA-PKcs and PARP blockage using Nu7026 and olaparib in cells containing chromosomally integrated HR-NHEJ reporter cassette. Hep3B cells containing the chromosomally integrated HR-NHEJ reporter cassette were pretreated with Nu7026 at

5 μM or/and olaparib at 10 μM for 24 h. Then cells were transfected with vectors encoding I-SceI and mTagBFP2. Inhibitors were added to the re-seeded cells post transfection. On day 2 post transfection, cells were harvested for FACS analysis. All experiments were repeated at least three times. Error bars represent s.d., ** $P < 0.01$, *** $P < 0.001$, n.s. not significant.

Supplementary Figure 3. (A) The transcription level of the reporter cassette in the 17 cell lines with the dual construct integrated at different loci. Exponentially growing cells were harvested for RNA extraction, followed by cDNA synthesis and q-PCR analysis of the transcription level. The primers used for PCR are as follows: Forward-CTGACCCTGAAGTTCATCTGCACC, Reverse-TTGAAGAAGATGGTGCGCTCCTG. **(B)** Analysis of the cell cycle distribution of the 9 cell lines with similar transcriptional level of the reporter. The 9 cell lines were harvested for cell cycle analysis on day 2 post splitting. **(C)** The differences in HR and NHEJ efficiency between chromosomal sites are not due to the difference in I-SceI cutting efficiency. The 7 cell lines containing the reporter cassette at different locations with comparable transcriptional level of the reporter and similar cell cycle distribution were transfected with 5 μg control vector pHPRT-CAG32 or I-SceI vector. At 2 h post transfection, cells were harvested for DNA and protein extraction. Genomic DNA was extracted from cells transfected with pHPRT-CAG32 or pCMV-I-SceI. Real time PCR with primers 5'AGGACAAACTCTTCGCGGTCTTTC3' and 5'CCTCGCCCTTGCTCACAAGCTTTAG3' amplifying across the second I-SceI recognition site, resulting in part of Ad2 exon and part of tdTomato ORF, was performed. Part of GAPDH gene amplified by primers 5'TGGTATGACAACGAATTTGG3' and 5'TCTACATGGCAACTGTGAGG3' was used as a control. At 2 hour post I-SceI transfection, when most of digested DNA has not been repaired by NHEJ or HR, the digested template does not allow for successful PCR amplification, leading to a decreased signal on real time PCR machine. Using comparative CT Method ($2^{-\Delta\Delta CT}$) the ratio of intact reporter cassette in I-SceI vector transfected cells/that in control vector transfected cells was determined. Then the cutting efficiency was calculated by subtracting the undigested ratio with 100%. All experiments were repeated at least three times. Error bars represent s.d.

Supplementary Figure 4. (A-D) The correlations between HR efficiency (tdTomato+/mTagBFP2+), NHEJ efficiency (GFP+/mTagBFP2+) and the densities of pre-existing nucleosomes at DNA damage sites (R1 and R2 regions). **(E-I)** The correlations between the choice of the two pathways HR/NHEJ, HR efficiency normalized to transcription level, NHEJ efficiency normalized to transcription level, HR efficiency, NHEJ efficiency and the densities of pre-existing nucleosomes at R3 are insignificant. **(J)** Change of nucleosome density at R2, R3, R4 and R5 regions at different time points post I-SceI transfection. All experiments were repeated at least three times. Error bars represent s.d., *** $P < 0.001$.

Supplementary Figure 5. (A) Western blot analysis of PARP1 depletion using two shRNAs against PARP1. **(B)** Representative FACS traces for the analysis of NHEJ and HR in control and PARP1 depleting D4a cells. **(C)** Inhibiting PARP1 by PJ34 suppresses HR but not NHEJ. **(D)** Schematic diagram of the HR reporter. The cell line HCA2-H15c harboring the HR reporter is as previously described (3,4). The reporter contains two inactivated GFP-pem1. Upon the induction of DSBs by I-SceI digestion at the 1st exon of the 1st copy of GFP-pem1, only HR can restore the functional GFP.

(E-G) Depleting PARP1 (E) or inhibiting PARP1 with olaparib (F) or PJ34 (G) significantly suppresses HR directed repair. **(H)** PAR chain formation in cells arrested in G1 or S stage following IR treatment. All experiments were repeated at least three times. Error bars represent s.d., * P < 0.05, ** P < 0.01.

Supplementary Figure 6. Olaparib sensitizes cancer cells but not normal cells to etoposide at low concentrations. The HR proficient cancer cell lines including MCF7, Hep3B and HeLa, and two immortalized normal cell lines MCF10A and Chang liver with low HR capacities were treated with both olaparib and etoposide at an increasing concentration. On Day 10 post drug treatment, colonies were stained and counted.

Supplementary Figure 7. (A) Representative images of RPA2 recruitment to radiation-induced DSBs upon PARP1 inhibitor olaparib or PJ34 treatment. The cells were co-immunostained for RPA2 (red), Geminin (green) and DAPI (blue). (**B)** Representative images of RAD51 recruitment to radiation-induced DSBs upon PARP1 inhibitor olaparib or PJ34 treatment. The cells were co-immunostained for RAD51 (green), Geminin (red) and DAPI (blue). **(C)** Neither Olaparib nor PJ34 reduced the proportion of cells in S/G2 stage, in which HR occurs. **(D)** HR is inhibited upon olaparib or PJ34 treatment at different concentrations. All experiments were repeated at least three times. Error bars represent s.d., * P < 0.05, ** P < 0.01, *** P < 0.001, n.s. not significant.

Supplementary Figure 8. (A-B) PARP1 and PAR do not interact with RPA2 in the absence or presence of DNA damages. Co-IP experiments were performed with an antibody against PARP1 or PAR and then WB against RPA2 was performed. **(C-D)** Raw data of relative nucleosome density in Fig. 3H-I. **(E)** Representative images of RPA2 recruitment to radiation-induced DSBs upon PARP1 inhibitor treatment with / without chloroquine pre-treatment. The cells were co-immunostained for RPA2 (red), Geminin (green) and DAPI (blue). **(F)** Pretreatment with VPA rescues the decline of HR in olaparib or PJ34 treated D4a cells. **(G)** PJ34 and Olaparib, two PARP1 inhibitors, have mild effect on PARP1 protein level. Cells were pretreated with PJ34 or Olaparib for 16 hours before being harvested for protein extraction followed by Western blot analysis.

Supplementary Figure 9. (A) PAR interacting proteins in response to IR stained with commassie blue on a SDS page gel. The pulled down proteins by an antibody against PAR were subjected to mass spec analysis. The list shows parts of PAR interacting proteins associated with DNA damage repair post IR. BRG1 and SIRT1 both have higher score and coverage than proteins that were previously shown interacting with PAR, such as Werner Syndrome RecQ Like Helicase (WRN) (6,7), or proteins that were parylated such as PARP1 itself, PARP4. **(B)** *In vitro* pull down analysis of the interaction between purified recombinant BRG1 and biotin-labeled PAR. The recombinant BRG1-His protein was purified from 293F cells and incubated with biotin-labeled PAR for 12 h at 4°C. Streptavidin beads were added to the reaction and incubated for 2 h at 4°C, followed by WB analysis with an antibody against His. **(C)** BRG1 is not parylated in response to DNA DSBs. The irradiated or unirradiated cells were harvested for co-IP analysis with an antibody against BRG1, followed by WB analysis with PAR antibody. **(D)** *In vitro* pull down analysis of the interaction between three purified recombinant BRG1 domains and biotin-labeled PAR.

Supplementary Figure 10. (A) Representative FACS traces for the analysis of NHEJ and HR in control and BRG1 depleting D4a cells. **(B)** BRG1 depletion impairs HR in HCA2-H15c cells harboring

the well-established HR reporter. **(C)** BRG1 depletion has no effect on cell cycle distribution. **(D)** Raw data of relative nucleosome density in Fig. 4H. **(E)** Pretreatment with VPA rescues the decline of HR in BRG1 depleted cells. **(F)** Raw data of relative nucleosome density in Fig. 4J. **(G)** Analysis of nucleosome sliding activity of BRG1 in the absence or presence of PARP1.

Supplementary Figure 11. (A) *In vitro* pull down analysis of the interaction between purified recombinant SIRT1 or SIRT1 (ΔZnF) and biotin-labeled PAR. **(B)** Representative FACS traces for the analysis of NHEJ and HR in control and SIRT1 depleting D4a cells. **(C)** SIRT1 depletion suppresses HR in HCA2-H15c cells harboring the well-established HR reporter. **(D-E)** Raw data of relative nucleosome density in Fig. 5H-I. **(F-G)** Epistasis analysis of PARP1 and SIRT1 effect on nucleosome density. (G) is the raw data of (F). **(H)** Epistasis analysis of PARP1 and SIRT1 effect on HR repair. Pretreatment with chloroquine rescues the reduction in HR efficiency in D4a cells with PARP1 inhibited by PJ34 and SIRT1 depleted. All experiments were repeated at least three times. Error bars represent s.d. ** $P < 0.01$, n.s. not significant.

Supplementary Figure 12. (A) *In vitro* biochemical reactions showing the BRG1-ATPase domain is deacetylated by SIRT1. **(B-C)** Lysine residues on BRG1 not affected by SIRT1. Predicted lysine residues which may be potentially deacetylated by SIRT1 (B). Co-IP analysis of acetylation levels of BRG1 WT and mutants (C).

Supplementary Figure 13. (A) Representative pictures of RPA2 recruitment to radiation-induced DSBs. The cells were co-immunostained for RPA2 (red), Geminin (green) and DAPI (blue). Pictures were then taken on a Nikon confocal microscope. **(B)** Representative pictures of RAD51 recruitment to radiation-induced DSBs. The cells were co-immunostained for RAD51 (green), Geminin (red) and DAPI (blue). Pictures were then taken on a Nikon confocal microscope.

Supplementary Figure 14. (A-B) Lolliplots show the distribution of mutations in BRG1. In lung cancer and pan-cancer cell line, BRG1 shows a high mutation rate (7.1% and 9.7%), and mutations which may affect protein function have a strong tendency to occur in ATPase domain in both pan-cancer cell lines (A) and lung cancers (B).

Supplementary Figure 15. The model for the PAR-SIRT1-BRG1 mediated DNA DSB repair by HR.

Supplementary Table 1. Genomic positions of the HR-NHEJ reporter cassette in the HCA2-hTERT cell lines. The genomic DNA of all the cell lines were extracted and genome walking was performed to identify the positions of the reporter cassette.

REFERENCES

1. Anglana, M. and Bacchetti, S. (1999) Construction of a recombinant adenovirus for efficient delivery of the I-SceI yeast endonuclease to human cells and its application in the in vivo cleavage of chromosomes to expose new potential telomeres. *Nucleic acids research*, **27**, 4276-4281.
2. Seluanov, A., Mittelman, D., Pereira-Smith, O.M., Wilson, J.H. and Gorbunova, V. (2004) DNA end joining becomes less efficient and more error-prone during cellular senescence.

Proceedings of the National Academy of Sciences of the United States of America, **101**, 7624-7629.
3. Mao, Z., Seluanov, A., Jiang, Y. and Gorbunova, V. (2007) TRF2 is required for repair of nontelomeric DNA double-strand breaks by homologous recombination. *Proceedings of the National Academy of Sciences of the United States of America*, **104**, 13068-13073.
4. Mao, Z., Bozzella, M., Seluanov, A. and Gorbunova, V. (2008) Comparison of nonhomologous end joining and homologous recombination in human cells. *DNA repair*, **7**, 1765-1771.
5. Tang, J., Cho, N.W., Cui, G., Manion, E.M., Shanbhag, N.M., Botuyan, M.V., Mer, G. and Greenberg, R.A. (2013) Acetylation limits 53BP1 association with damaged chromatin to promote homologous recombination. *Nature structural & molecular biology*, **20**, 317-325.
6. Khadka, P., Hsu, J.K., Veith, S., Tadokoro, T., Shamanna, R.A., Mangerich, A., Croteau, D.L. and Bohr, V.A. (2015) Differential and Concordant Roles for Poly(ADP-Ribose) Polymerase 1 and Poly(ADP-Ribose) in Regulating WRN and RECQL5 Activities. *Molecular and cellular biology*, **35**, 3974-3989.
7. Popp, O., Veith, S., Fahrer, J., Bohr, V.A., Burkle, A. and Mangerich, A. (2013) Site-specific noncovalent interaction of the biopolymer poly(ADP-ribose) with the Werner syndrome protein regulates protein functions. *ACS chemical biology*, **8**, 179-188.
