# Supplementary material for: A PARP1–BRG1–SIRT1 axis promotes HR repair by reducing nucleosome density at DNA damage sites
Source: Nucleic Acids Res. 2019 Jul 10;47(16):8563–80. doi: 10.1093/nar/gkz592 (PMC7145522; doi:10.1093/nar/gkz592)
Supplement: gkz592_Supplemental_Files [file gkz592_supplemental_files.zip › Supplementary Figure.pdf]

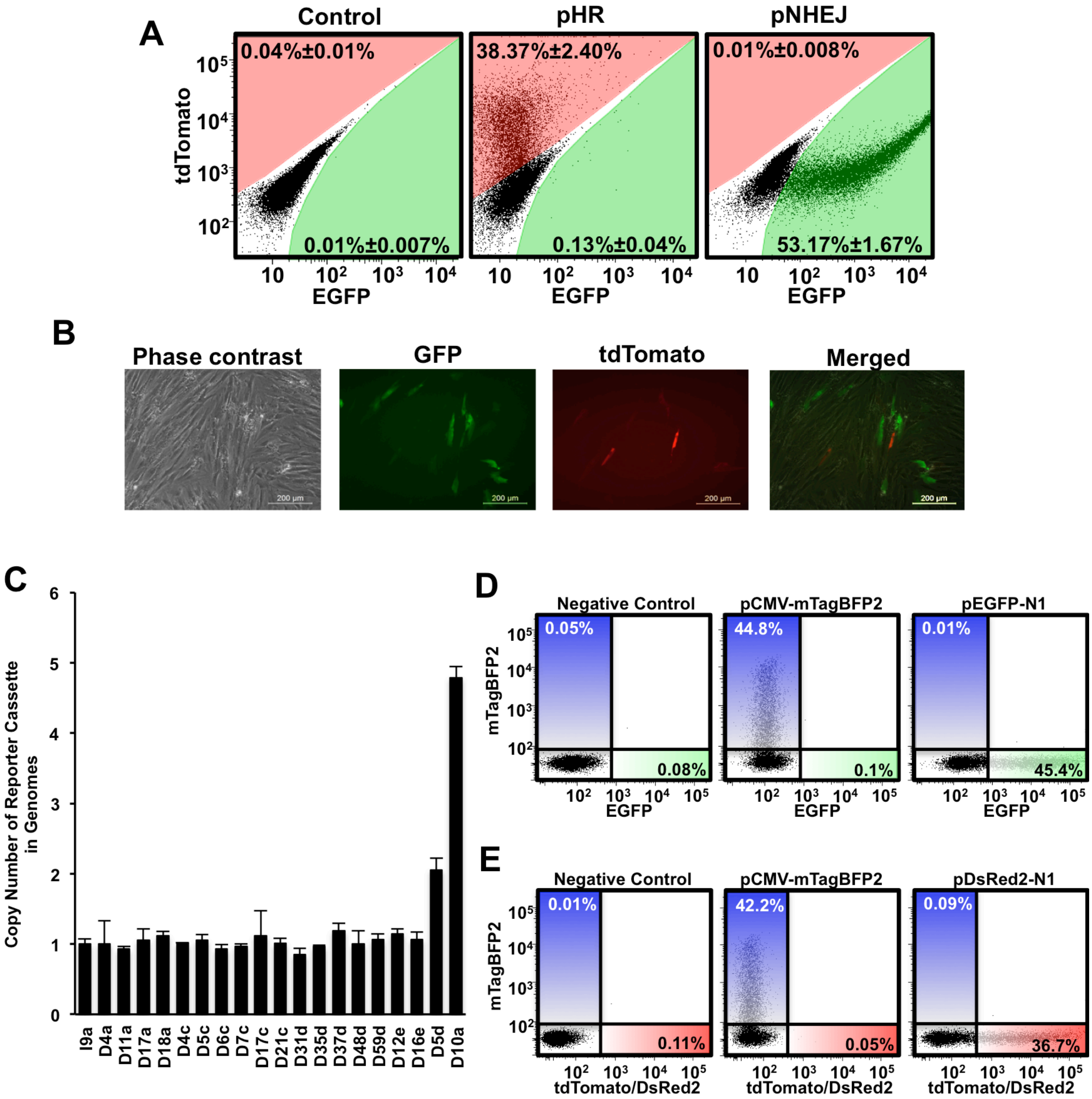

Supplementary Figure 1

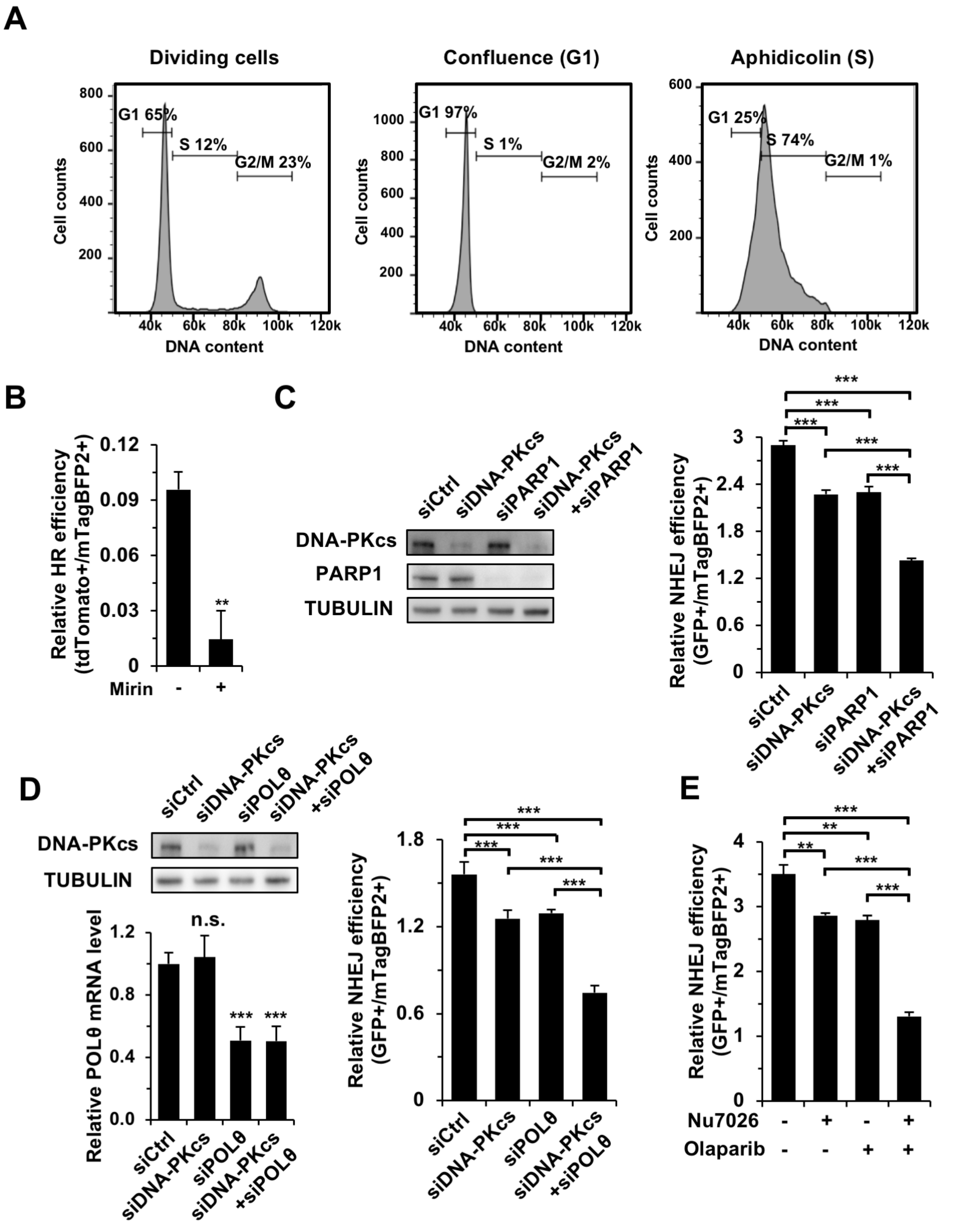

Supplementary Figure 2

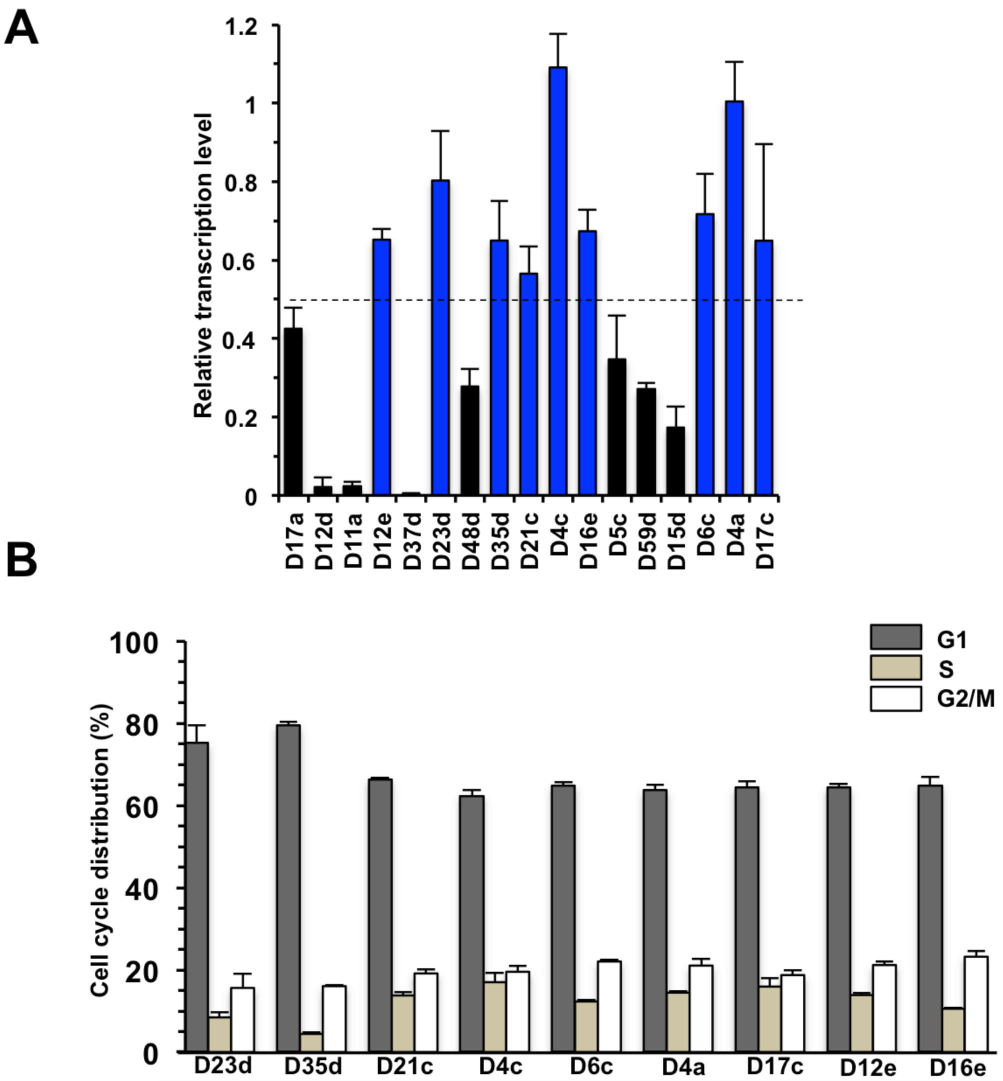

S/G2/M	24.03± 4.75	20.44± 0.72	32.90 ±1.71	36.53± 3.70	34.33± 0.80	35.50 ±1.88	34.67± 3.07	35.03± 1.46	33.73± 1.60

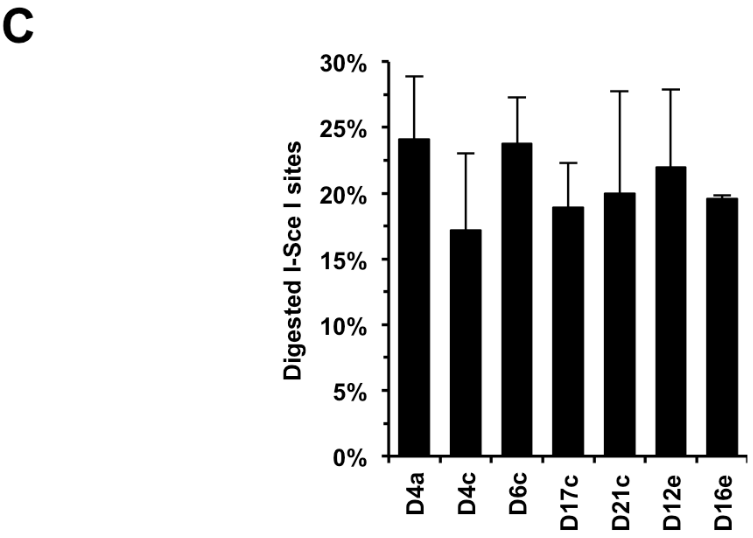

Supplementary Figure 3

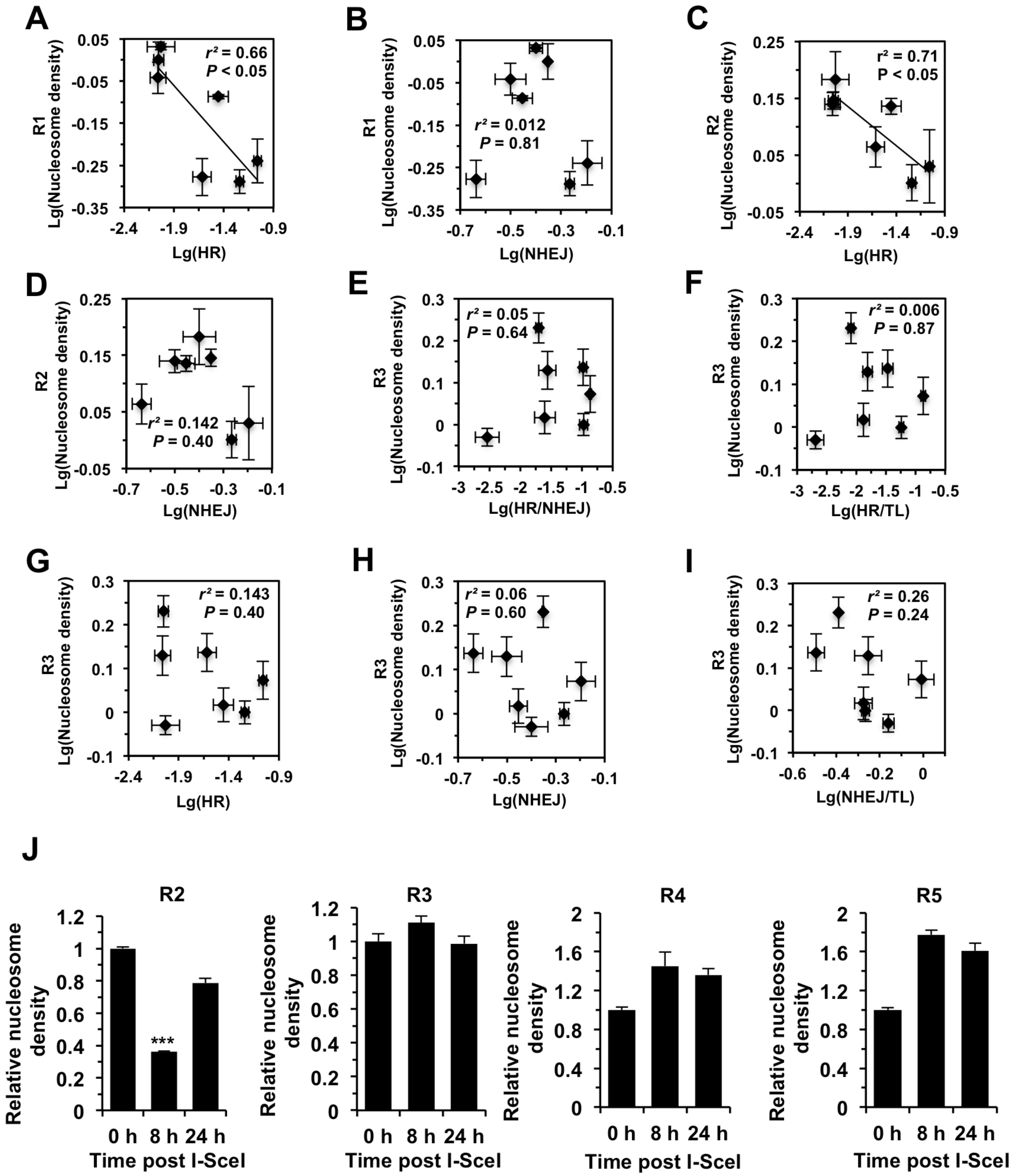

Supplementary Figure 4

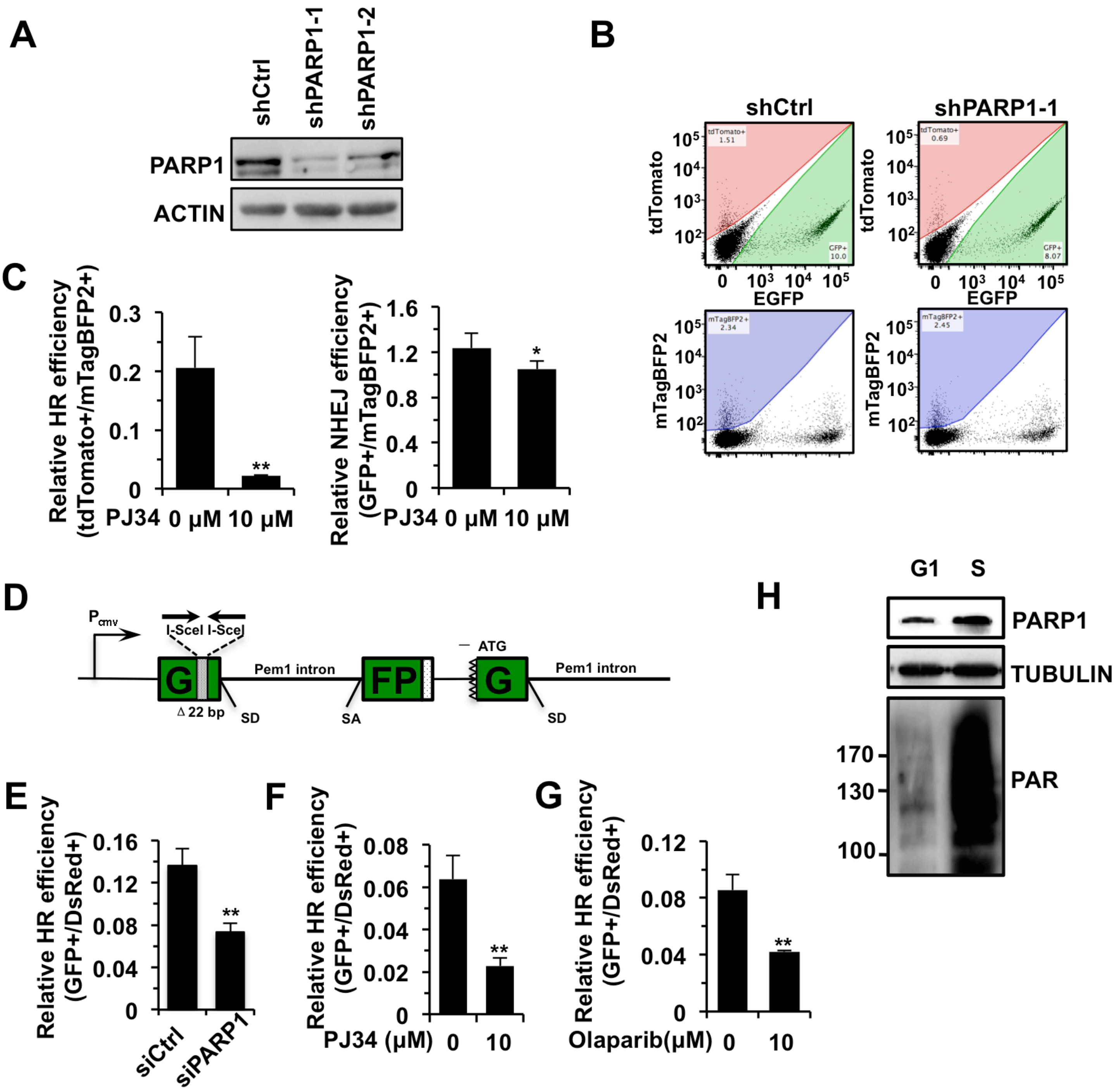

Supplementary Figure 5

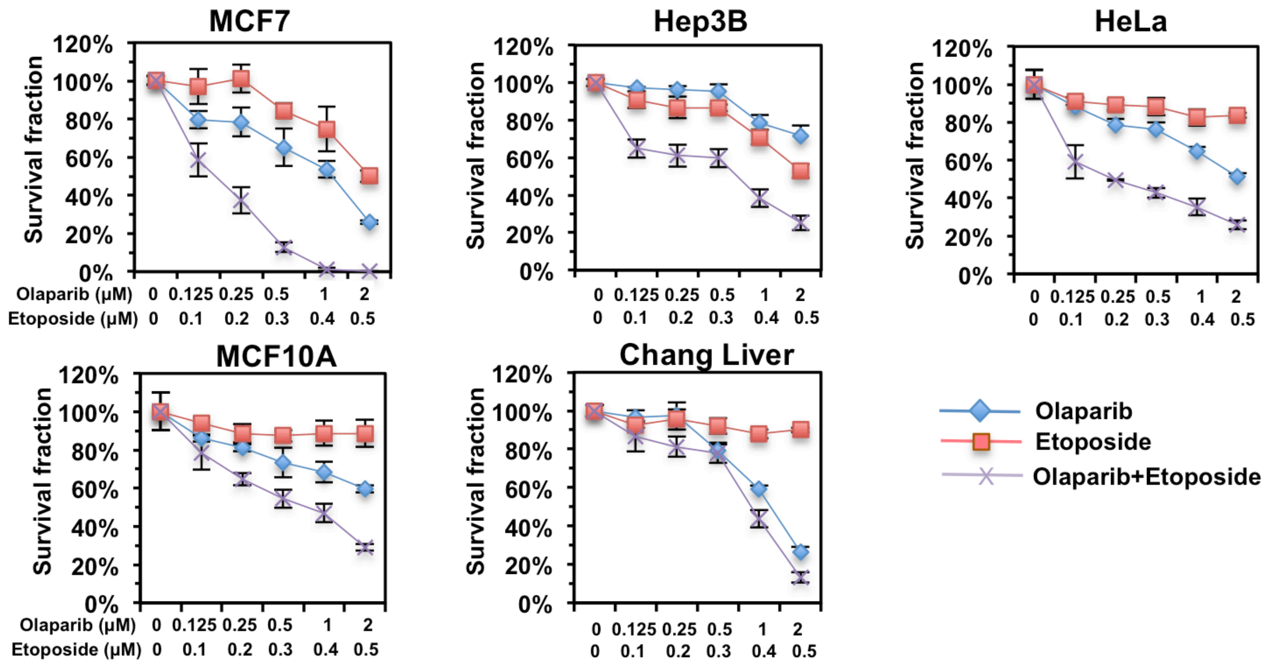

Supplementary Figure 6

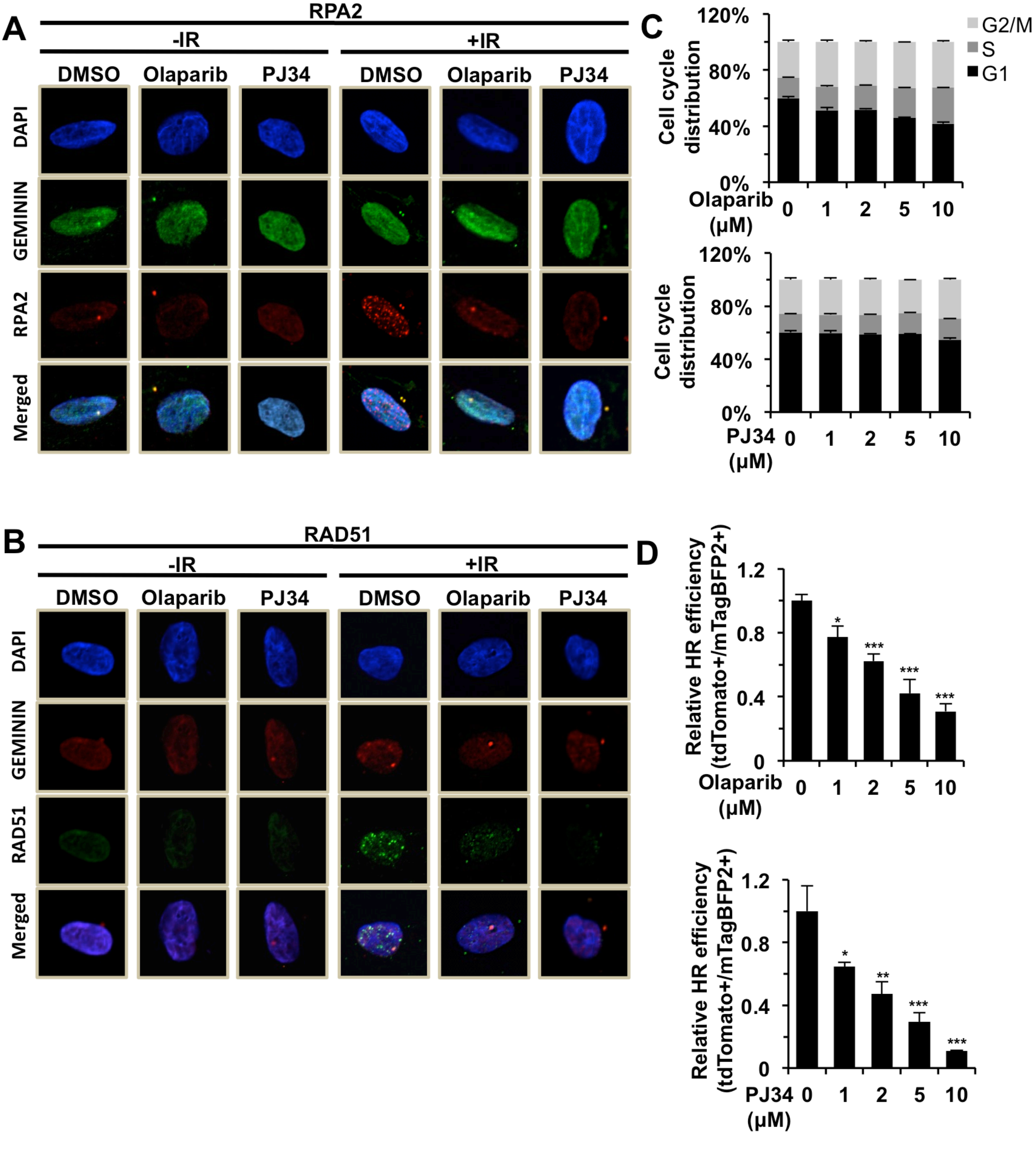

Supplementary Figure 7

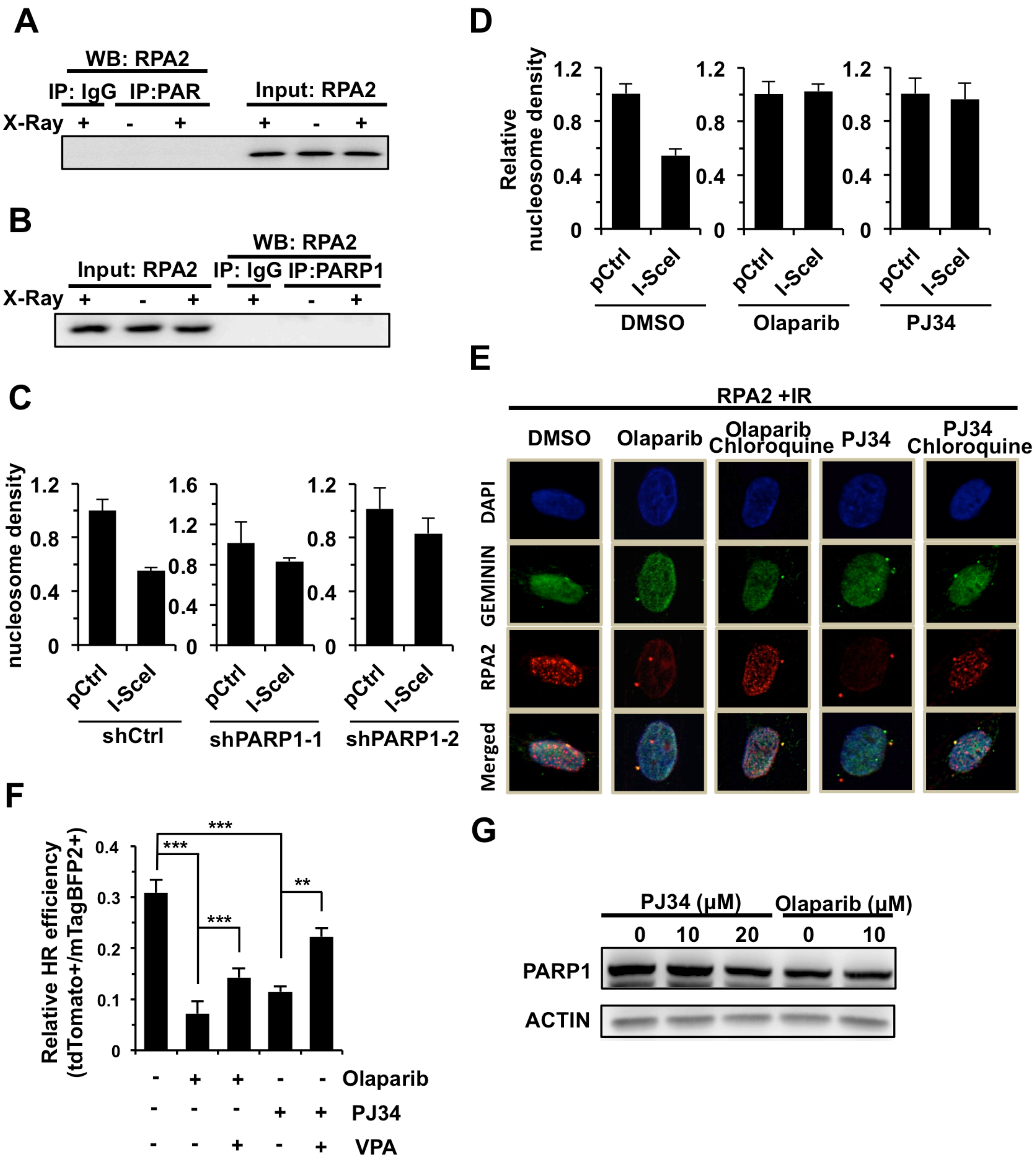

Supplementary Figure 8

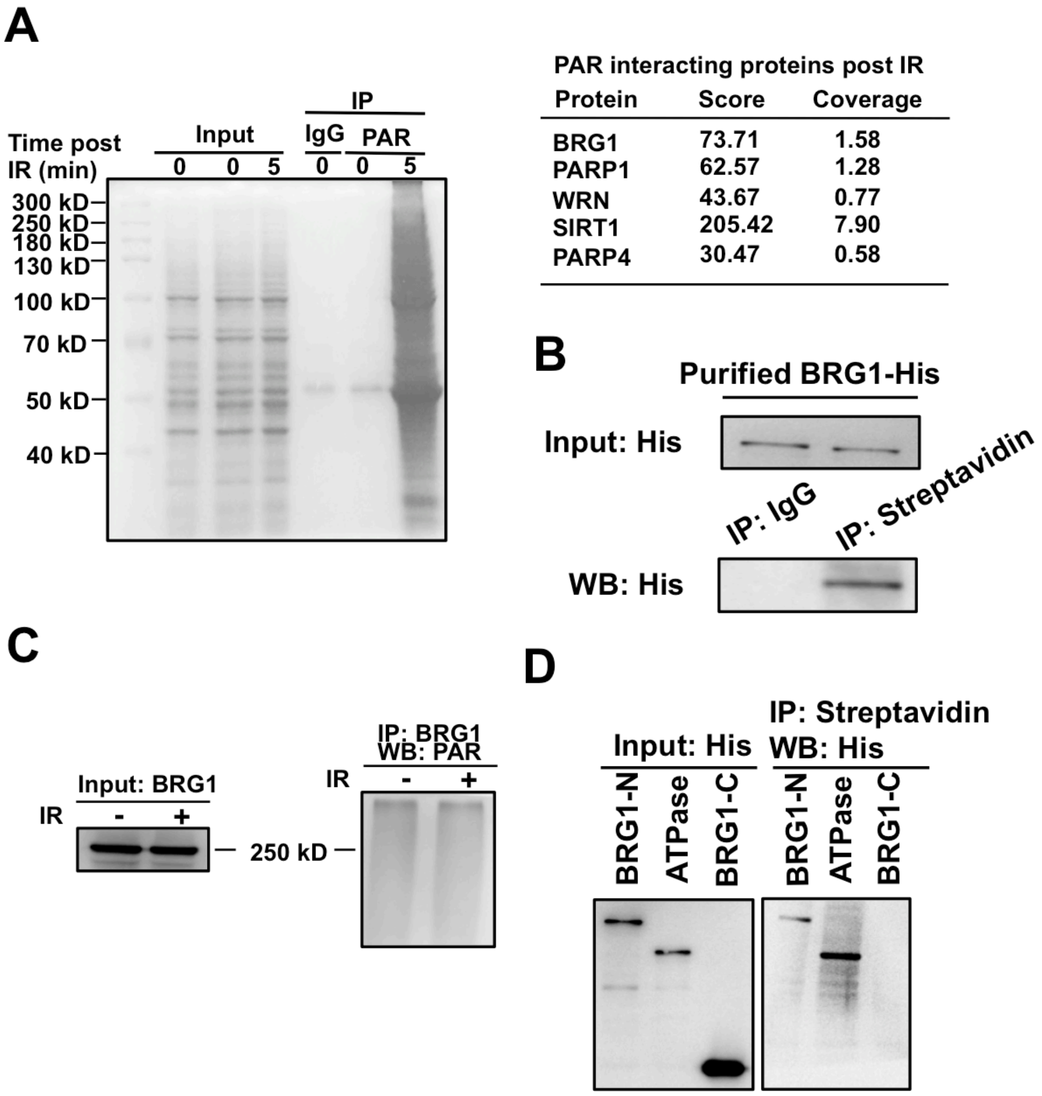

PAR interacting proteins post IR

Protein	Score	Coverage
BRG1	73.71	1.58
PARP1	62.57	1.28
WRN	43.67	0.77
SIRT1	205.42	7.90
PARP4	30.47	0.58

Supplementary Figure 9

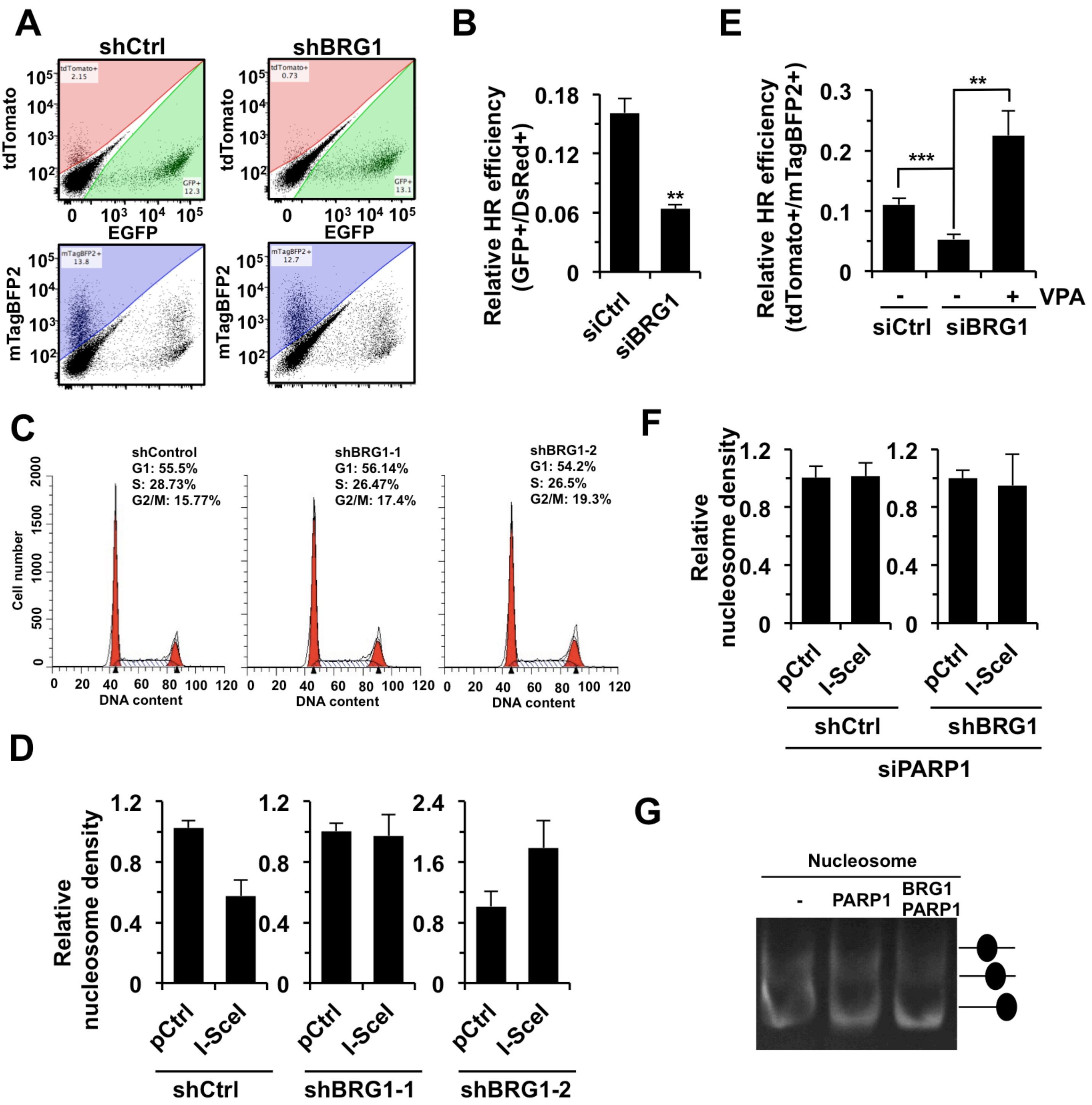

Supplementary Figure 10

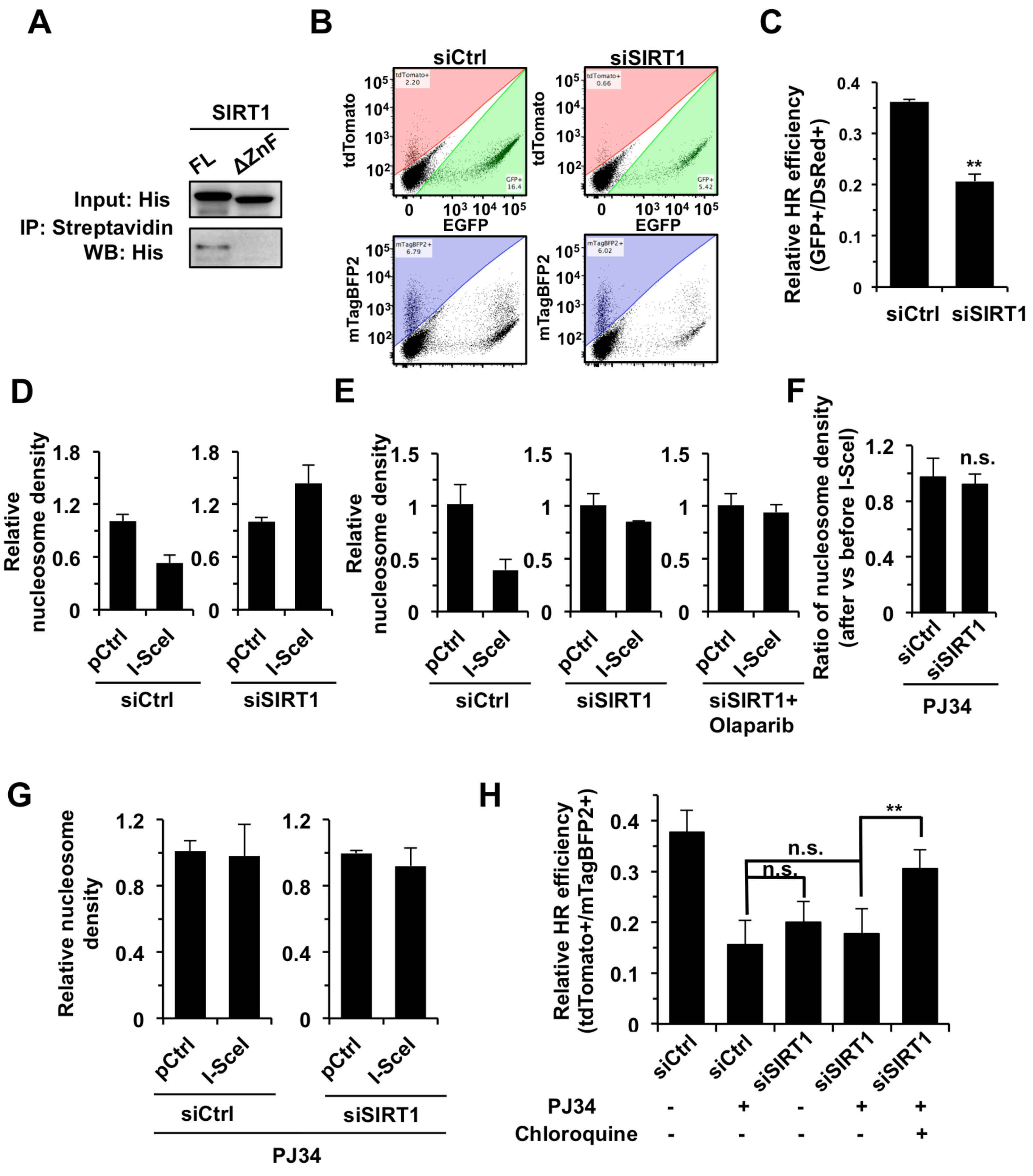

Supplementary Figure 11

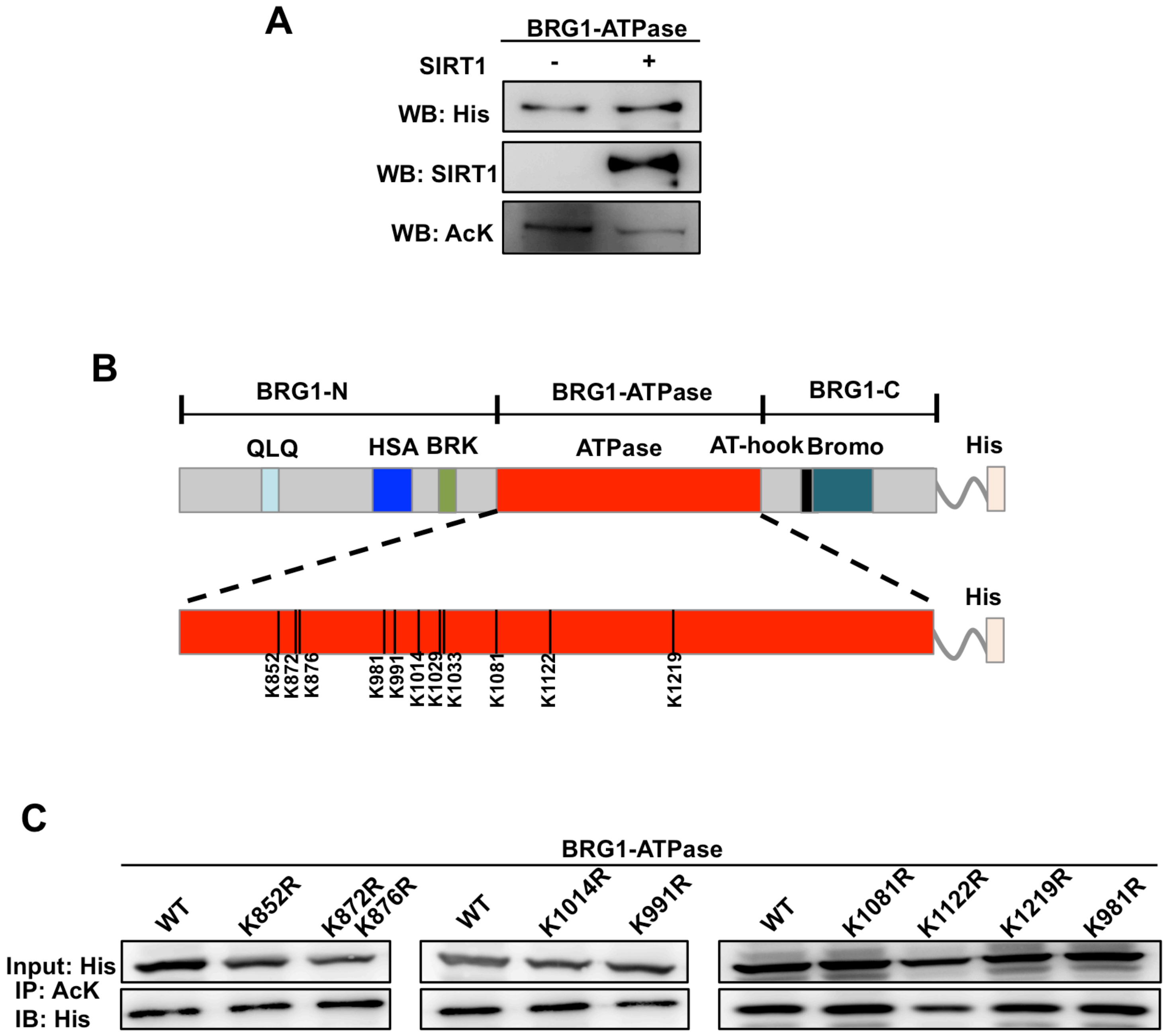

Supplementary Figure 12

A

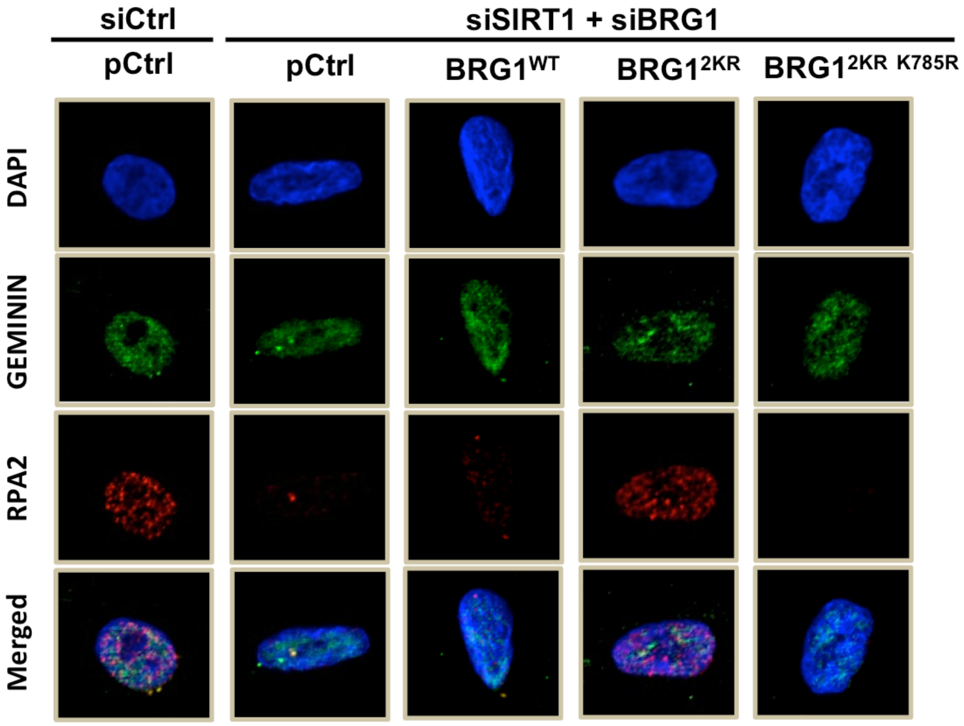

B

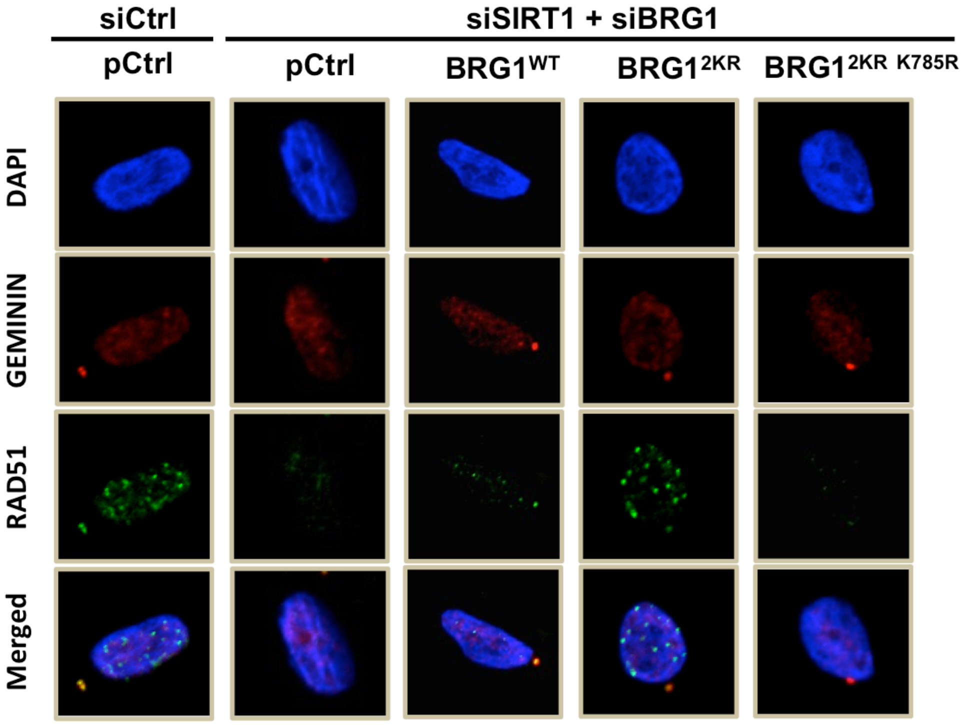

Supplementary Figure 13

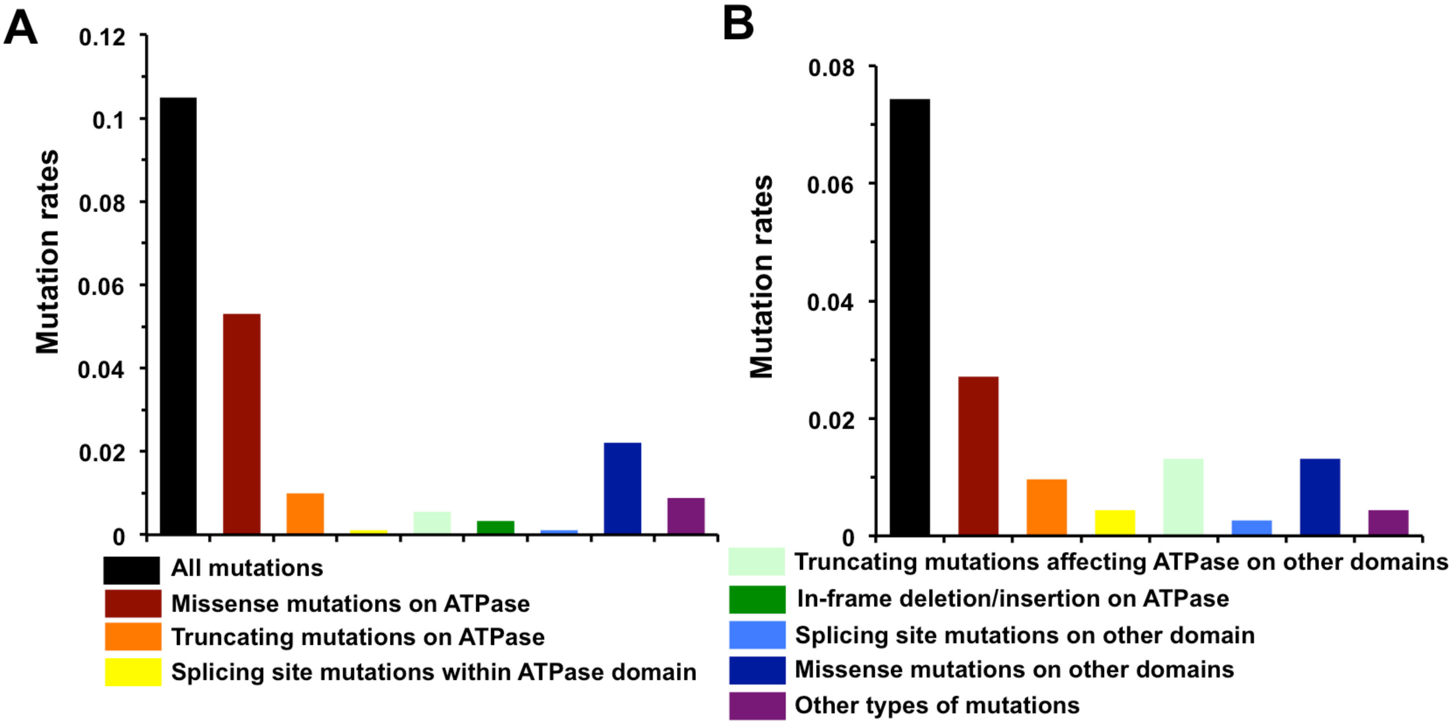

Supplementary Figure 14

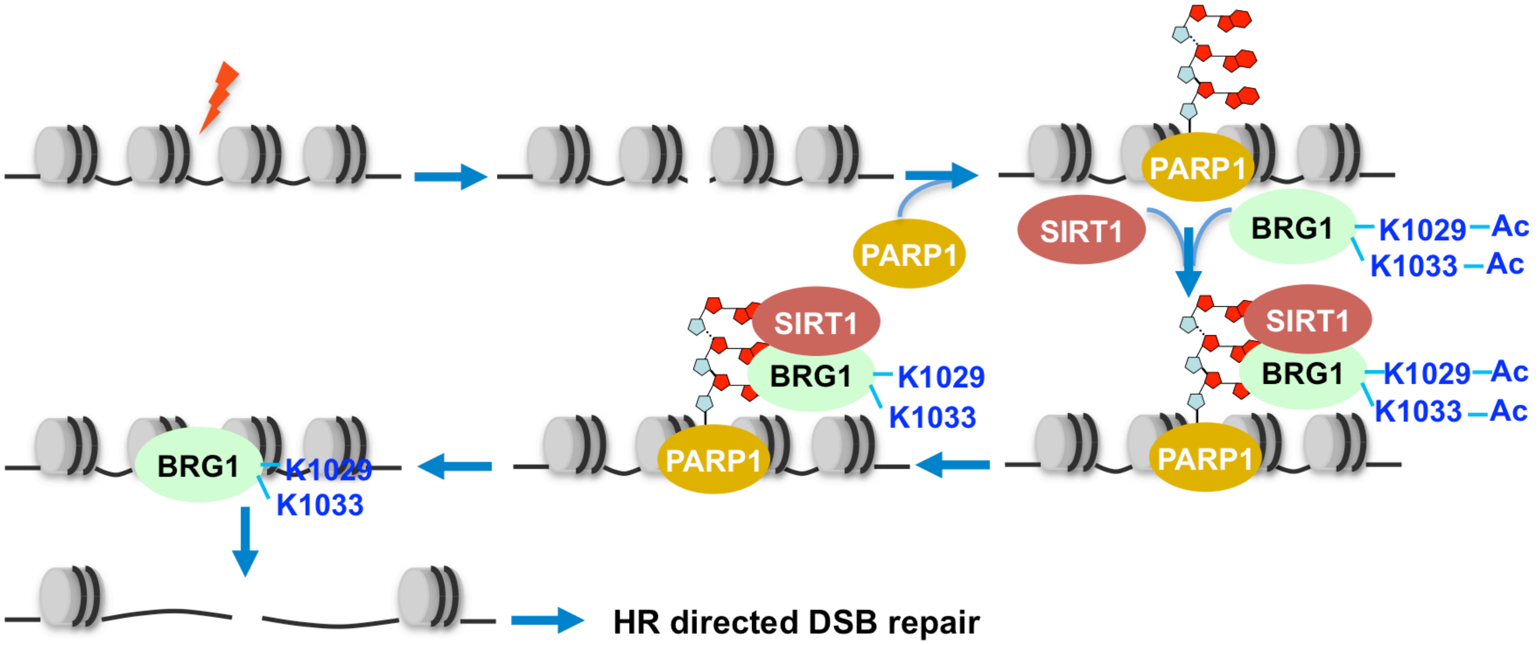

Supplementary Figure 15
