# Supplementary material for: A PARP1–BRG1–SIRT1 axis promotes HR repair by reducing nucleosome density at DNA damage sites
Source: Nucleic Acids Res. 2019 Jul 10;47(16):8563–80. doi: 10.1093/nar/gkz592 (PMC7145522; doi:10.1093/nar/gkz592)
Supplement: gkz592_Supplemental_Files [file gkz592_supplemental_files.zip › Supplementary Table.pdf]

Table. S1. Genomic positions of the HR-NHEJ reporter cassette in the HCA2-hTERT cell lines. The genomic DNA of all the cell lines were extracted and genome walking was performed to identify the positions of the reporter cassette.

Cell Line	Chromosomes	Positions on the chromosome	Nearest Gene
D4a	2	231349785	SP100
D11a	9	37348875	ZCCHC7
D17a	X	133631148	HPRT1
D4c	12	46032656	BC053951
D5c	10	33528949	NRP1
D6c	13	74723877	KLF12
D17c	12	76675161	BBS10
D21c	6	125521797	TPD52L1
D35d	20	50017625	NFATC2
D37d	21	32469836	TIAM1
D48d	16	8968538	CARHSP1
D59d	5	58971658	PDE4D
